# Fault-Tolerant Trust-Based Task Scheduling Algorithm Using Harris Hawks Optimization in Cloud Computing

**DOI:** 10.3390/s23188009

**Published:** 2023-09-21

**Authors:** Sudheer Mangalampalli, Ganesh Reddy Karri, Amit Gupta, Tulika Chakrabarti, Sri Hari Nallamala, Prasun Chakrabarti, Bhuvan Unhelkar, Martin Margala

**Affiliations:** 1 School of Computer Science and Engineering, VIT-AP University, Amaravati 522237, India; ganesh.reddy@vitap.ac.in; 2 Department of ECE, Nalla Malla Reddy Engineering College, Hyderabad 500088, India; amitgupta.ece@nmrec.edu.in; 3 Department of Chemistry, Sir Padampat Singhania University, Udaipur 313601, India; tulika.chakrabarti@spsu.ac.in; 4 Vasireddy Venkatadri Institute of Technology, Nambur 522510, India; 5 Department of Computer Science and Engineering, Sir Padampat Singhania University, Udaipur 313601, India; 6 Muma School of Business, University of South Florida, Sarasota-Manatee, FL 33620, USA; bunhelkar@usf.edu; 7 School of Computing and Informatics, University of Louisiana at Lafayette, Lafayette, LA 70504, USA; martin.margala@louisiana.edu

**Keywords:** availability, Harris hawks optimization, rate of failures, SLA-based trust parameters, success rate

## Abstract

Cloud computing is a distributed computing model which renders services for cloud users around the world. These services need to be rendered to customers with high availability and fault tolerance, but there are still chances of having single-point failures in the cloud paradigm, and one challenge to cloud providers is effectively scheduling tasks to avoid failures and acquire the trust of their cloud services by users. This research proposes a fault-tolerant trust-based task scheduling algorithm in which we carefully schedule tasks within precise virtual machines by calculating priorities for tasks and VMs. Harris hawks optimization was used as a methodology to design our scheduler. We used Cloudsim as a simulating tool for our entire experiment. For the entire simulation, we used synthetic fabricated data with different distributions and real-time supercomputer worklogs. Finally, we evaluated the proposed approach (FTTATS) with state-of-the-art approaches, i.e., ACO, PSO, and GA. From the simulation results, our proposed FTTATS greatly minimizes the makespan for ACO, PSO and GA algorithms by 24.3%, 33.31%, and 29.03%, respectively. The rate of failures for ACO, PSO, and GA were minimized by 65.31%, 65.4%, and 60.44%, respectively. Trust-based SLA parameters improved, i.e., availability improved for ACO, PSO, and GA by 33.38%, 35.71%, and 28.24%, respectively. The success rate improved for ACO, PSO, and GA by 52.69%, 39.41%, and 38.45%, respectively. Turnaround efficiency was minimized for ACO, PSO, and GA by 51.8%, 47.2%, and 33.6%, respectively.

## 1. Introduction

Cloud computing is one of the rapidly growing technologies which impact the IT industry, causing companies to migrate enterprise infrastructures onto cloud environments as they deliver services to their customers around the world based on a pay-per-use basis, which offers flexibility to customers to reduce operational and management overhead for their enterprises [1]. This paradigm is also advantageous to the users in terms of flexibility in using resources of the cloud environment, and it makes use of scaling techniques [2] to increase or decrease resources based on the needs of customers and their usage in the application environment. Therefore, the cloud computing paradigm is advantageous for the users running their applications in a cloud environment, but to provide virtual resources to users from the cloud provider according to the respective Service Level Agreements, cloud providers need to use a task scheduler which provisions these resources based on customer needs. Every cloud provider has users around the globe, and it is difficult for the cloud provider to tackle user needs by provisioning virtual resources manually; therefore, a task scheduler is needed, one which maps incoming tasks from various users to the cloud interface, mapping them according to appropriate VMs. Therefore, a task-scheduling algorithm is needed which maps a variable number of tasks to appropriate virtual resources to benefit both cloud providers and users. In the cloud paradigm, task scheduling is a challenging scenario, as incoming tasks placed onto the cloud platform are variable as per their sizes and run-time processing capacities. We cannot specify a number of tasks to be fixed in the cloud paradigm; moreover, the tackling and mapping of these tasks to virtual resources by providing high availability and a fault-tolerance system to users is a challenge from the cloud provider’s perspective, because single-point failures [3] still occur in the cloud computing paradigm, which impacts the fault tolerance of the system as well as trust in the cloud provider. This motivates us to perform the present research from the perspective of fault tolerance and trust in the cloud computing paradigm. Many of the earlier authors developed various task schedulers by using nature-inspired, meta-heuristic approaches such as swarm intelligence algorithms and bio-inspired algorithms, as task scheduling in cloud computing is an NP-hard problem, and it is difficult to identify a solution in the specified polynomial amount of time. Many of the existing authors used PSO [4], GA [5], and ACO [6], as well as their variants, but the question is not limited to these approaches, and each of them has their advantages, while there are some limitations manifest in their approaches. Existing authors have tackled various parameters, i.e., makespan, execution cost, throughput, etc., but no author has tackled parameters with fault-tolerance and trust-based parameters. In this research paper, we focused on developing a task scheduler that considers tasks and VM priorities and schedules tasks using Harris hawks optimization while addressing certain parameters, i.e., turnaround efficiency, failure ratio, availability of VMs, and success rate.

### Motivation and Contributions

The cloud-computing model delivers services on demand to all cloud users. All cloud providers have wide varieties of services, and these services are delivered to customers around the world based on the requirements of the users. In order to provide seamless access to all users, an effective task-scheduling mechanism should be used by cloud providers, while they also need to be concerned about high availability and fault tolerance in cloud computing. In this model, single points of failure still persist, which impacts QoS and violates the SLA, in turn ruining trust in the cloud provider. This motivates us to conduct work in this direction to tackle the parameters, i.e., fault tolerance and SLA-based trust parameters. We also identified a relationship between fault tolerance and trust in the cloud provider, i.e., the presence of a minimal number of task failures increases trust as well as the QoS of the cloud provider. To retain good quality of the services provided to cloud users and to gain trust in the cloud provider by minimizing SLA-based parameters, we decided to design a task scheduler that is modelled using Harris hawks optimization. Highlights of this research are mentioned below.

- The aim is to design a fault-tolerant trust-based task scheduling algorithm by mapping all incoming tasks to VMs using Harris hawks optimization.
- For an effective scheduling process, the carefully calculated priorities of all incoming tasks should be accurately mapped to appropriate and low-electricity-unit-cost VMs by calculating VM priorities. Task-level and VM-level priorities are calculated to schedule tasks precisely onto respective VMs.
- We introduce a deadline constraint into our scheduler to carefully assign a single task to a VM at a time, and after completion of that task within the stipulated time, the next task can be assigned to that respective VM.
- We conduct workload generation in two phases. In the first phase, we use random generated workload employing different statistical distributions. In the second phase, we use real-time computing cluster worklogs.
- In this research work, we address parameters such as failure rate, makespan, success rate, availability and turnaround efficiency.

Our manuscript is organized as follows. Section 2 discusses related works, Section 3 describes fault-tolerant trust-based task scheduling and system architecture, Section 4 describes simulation and results, and Section 5 discusses conclusion and future works.

## 2. Related Works

Load balancing and resource utilization are crucial factors in the cloud paradigm; they directly impact the makespan of tasks, but the balancing of tasks while utilizing resources in a wise manner is a huge challenge in this paradigm. Therefore, the authors in [7] formulated an adaptive task-scheduling mechanism which tackles the above-mentioned problem. They used an LDIW technique in combination with a famous swarm intelligence approach, i.e., PSO. The LDIW technique balances both local and global search, which explore the search space in a rigorous manner. It was evaluated over various existing techniques and from the extensive simulation results that LDIW improves makespan and throughput compared to existing techniques by 10% and 12% respectively. In the cloud paradigm, many researchers used metaheuristic or swarm intelligence algorithms to model the scheduling algorithms as it is an NP-hard problem. The authors in [8] used a technique that was added to PSO to schedule a longest task to a fastest processor in combination with PSO, minimum-completion-time jobs to different processors by classifying tasks to address the makespan, and total execution time. The method was compared with state-of-the-art approaches. The results showed huge improvement in performance of various parameters mentioned over the existing algorithms. Utilization of virtual resources and makespan are primary metrices in task scheduling, and in that regard, the authors in [9] used a hybridized approach to explore the search space and tackle the scheduling problem in the cloud paradigm in a comprehensive manner. For this, they used GA and GELS algorithms to model the scheduler. Extensive simulations were carried out using MATLAB2018a. The model was evaluated over baseline approaches: GA, PSO and GELS. Simulation results revealed that the hybrid model, i.e., GAGELS, improved the above-mentioned parameters compare to the state-of-the-art algorithms. The authors in [10] developed a load balancing technique to maximize utilization of virtual resources. This approach modelled the use of a modified PSO by collecting all the tasks and VM information carefully to schedule tasks onto VMs. Experiments were conducted on Cloudsim. The model was compared against the existing algorithms, such as PSOBTS, L-PSO, DLBA. Results showed that MPSO dominates all other approaches in the above-specified parameters. The above-mentioned algorithms are mentioned [7,8,9,10] in regard to makespan and resource utilization, but these are primary concerns for any task scheduler. In the next part of the paper, we discuss other parameters including makespan and resource utilization.

The authors in [11] formulated a task-scheduling approach for scientific workflows as it is difficult to identify dependencies in it. For the modelling of this approach, they used two strategies, i.e., scheduling and data placement approaches. The main aim of this approach is to precisely schedule workflows onto virtual resources while minimizing data movements in-between datacentres. IT2FCM is a Fuzzy method used as a methodology to design a scheduler. MATLAB2018a was used as a simulation tool for conduction of extensive experiments. The model was compared with the existing state-of-the-art approaches. Results revealed that it shows dominance over existing scientific workflows for minimization of data movements in-between datacentres. The authors in [12] formulated a deadline-aware task-scheduling mechanism for computational intensive, independent tasks, tackling multiple objectives by optimizing time and cost. This approach schedules tasks dynamically based on resource capacity. It was modelled by modifying PSO. It was evaluated over the existing variations of PSO. Results proved that PSO-RDAL dominates and improves parameter makespan, response time, deadline of tasks, and execution costs. A multi-objective task scheduling model was developed in [13] to tackle makespan, processing cost, and resource utilization. This approach was modelled by hybridization of PSO and chaotic algorithms to avoid local optimum and to gain better convergence towards solution. Therefore, PSO was enhanced using sinusoidal, Lorenz iterators by a chaotic algorithm to map tasks to precise VMs. EPSOCHO uses standard benchmark worklogs to check the efficacy of an algorithm, and the results showed impact on existing approaches for mentioned parameters. The authors in [14] proposed a task-scheduling algorithm in IaaS clouds for a large scale of tasks. In this approach, the authors used the modified symbiotic search optimization technique by using a simplified mutualism process by using variations in respective arithmetic and geometric means of mutual vectors in the process of generation of next-generation solutions. The Cloudsim 3.0.3 simulator was used for experimentation, and it generated tasks ranging from 100 to 1000 for 40 iterations. It was compared to existing SOS and PSO-SA in regard to minimization of the makespan. Simulated results proved that GSOS outperforms other variations for the makespan by minimizing it to 20%. For the cloud provider, it is difficult to map a large number of exponential increase in heterogeneous user tasks to various heterogeneous resources unless there is an intelligent task scheduler. For this, the authors in [15] formulated a task-scheduling algorithm to cut down costs and unnecessary usage of resources in the cloud environment. In this approach, a binary operator was used to place particles at certain places to search the solution space. The method was combined with neural networks to expedite the process. Cloudsim was used as simulating environment by considering standard configuration settings. In [11,12,13,14,15,16], the authors doscuss task scheduling with respect to the movement of data, task execution cost, penalty and the deadline of tasks. These are also crucial factors while scheduling tasks onto precise VMs so as to follow SLA accurately.

For the calculation of efficiency of AINN-BPSO, the model was compared against existing meta-heuristic approaches. Results revealed that AINN-BPSO surpasses baseline approaches for metrices such as makespan, degree of imbalance, and cost. When task scheduling is ineffective, it is challenging for the cloud provider to maintain quality of service while rendering services to users. Therefore, to preserve the scheduling efficiency, the authors in [16] devised a scheduling algorithm, QPSO, by modifying placement of particles in a quadratic shape in the solution space for a better convergence towards solutions. A Cloudsim toolkit was used as a platform to implement the scheduling process, and it was evaluated against baseline PSO. Results revealed that QPSO improves scheduling efficiency by 20%. Task transfer time is crucial in the cloud paradigm as the tasks are scheduled and computed in the cloud network. If a scheduler maps tasks to an unsuitable resource, the transfer time of tasks increases, which leads to a delay in task processing and responding to user requests. Therefore, to tackle this situation, it is necessary to generate a schedule to a precise resource. The authors in [17] used a multi-verse optimizer by combining the model with GA which schedules tasks to appropriate resources, and it also reschedules tasks if tasks are delayed or terminated based on weights assigned to tasks with respect to resources in the cloud, i.e., task capacity, length, speed, processing capacity of resources, and throughput. MATLAB2018a was used as a platform simulate the algorithm. After simulation, the model was compared with different variants of GA, and simulation results revealed that MVO-GA improves task transfer time significantly. The authors in [18] devised a task-scheduling mechanism to map diversified tasks to available resources in order to expedite the scheduling process while minimizing execution cost, time, and power consumption. NSGAIII was used to model a scheduling algorithm by modifying the population selection intensively and over a diversified space to obtain greater convergence towards solutions. It was evaluated in comparison to the NSGAII approach to test its efficacy, and the results proved that NSGAIII shows significant improvement in regard to the above-mentioned parameters. Load balancing also plays a prominent role in combination with the scheduling aspect in cloud computing, as it helps to improve the quality of service of the cloud provider. For this reason, the authors in [19] developed a load balancing mechanism using a hybridized approach. For proper selection of tasks and their precise mapping to VMs, a lion optimizer was used, and for the fine tuning of task selection and mapping onto precise VMs, GA was used in a global search space. The model was compared with baseline algorithms; hybrid lion–GA improves load balancing compared to GA and lion optimization approaches. In [20], the authors proposed a task scheduler to improve the efficiency of the task-scheduling process. The model was constructed by the GSAGA algorithm where GSA was used in local search exploration and GA was used in global search exploration. GSAGA was compared to different meta-heuristics, and finally, the results showed a better impact on scheduling efficiency compared to that of existing approaches by minimizing makespan to a larger extent. The authors in [21] proposed a task scheduler approach to improve the accuracy of scheduling. The GBO algorithm was used as a methodology to obtain greater convergence towards solutions for scheduling, and a round-off value-based strategy was also employed. The Cloudsim toolkit was used as a simulation platform and compared with existing GA and PSO approaches; results revealed that GBO surpasses GA and PSO in regard to the above-mentioned parameters.

Makespan and cost are the primary concerns in task scheduling for the cloud computing paradigm. To tackle these aspects, the authors in [22] devised a multi-objective hybrid scheduling model by modifying RDWOA with a mutation operator. This RDWOA was hybridized with the MBA approach, and it was used for the scheduling process. The mode; was compared with state-of-the-art algorithms like IWC, MALO, and MGGS. Simulation results proved that HWOA-MBA surpassed other algorithms by minimizing the above parameters. The authors in [23] also discussed the role of makespan and cost in the task-scheduling process in the cloud paradigm. Whale optimization is improved by modifying inertia weights according to the speed of whales to avoid premature convergence in local search process; for global search process, it is combined with wild horse optimization to expedite the process of scheduling. The model was simulated on the Cloudsim platform and evaluated over standard whale and horse optimization techniques. IWHOLF-TSC dominated the standard whale and horse optimizations in regard to minimization of makespan and cost. In [24], the authors discussed the minimization of makespan and cost in the task-scheduling process in the cloud paradigm. The authors modified standard ACO by adding appropriate weights to ants to explore the search space and to avoid premature convergence. An extensive set of experiments were conducted to assess makespan and cost, and the results were evaluated against ACO, QANA, MTF-BPSO, MM, and FCFS algorithm data. HWACOA minimized the above parameters over baseline approaches. Datacenter processing time is one of the crucial aspects in a cloud model as it impacts QoS and operational costs of a cloud provider. In [17,18,19,20,21,22,23,24], the authors again discussed load balancing, resource utilization, makespan, and cost, but the references mentioned previously, which tackle makespan and cost, were based on single-nature-inspired approaches, whereas the approaches mentioned in [17,18,19,20,21,22,23,24] are all hybridized approaches.

Generally, the scheduling of tasks is generated based on events, but it is only suitable for single objectives. In tackling multiple objectives, which is a complex process, generalized meta-heuristics are not providing accurate results. Therefore, a neural network is to be added to the existing ACO in [25] to explore the solution space for a recursive generation of schedules while tuning the parameters, i.e., response time, cost, and datacenter processing time. LBACO was compared to standard ACO, and it was found that it generated better schedules than standard ACO while minimizing the above-mentioned parameters. Consistency is an important criterion in task scheduling; many meta-heuristic approaches have been developed to tackle this problem. But scheduling is an NP-hard problem. It is difficult to identify solutions and converge towards solutions for any type of workload generated through various users. Therefore, the authors in [26] formulated a scheduling mechanism to address violations of SLA, resource utilization and makespan. A hybrid multi-objective technique, i.e., QOGSHO, was used as a methodology in this approach. An extensive set of simulations was carried out on the Cloudsim platform. QOGSHO revealed its efficacy of consistency in the scheduling pattern while minimizing violations of SLA and makespan and improving resource utilization. A multi-objective task scheduling mechanism was formulated to address schedule length and execution cost in [27]. To tackle scheduling in this paradigm, HHO was modified by elite-based learning strategy, i.e., ELHHO, in which opposition learning is used in the exploration phase. The model was compared to the baseline approach, i.e., opposition learning and Harris hawks optimization, and results showed that ELHHO outperforms existing mechanisms for the above-mentioned parameters to a great extent. Reliability is an important facet in task scheduling because even if a scheduler generates schedules over resources, if resource reliability is not preserved, then problems in the cloud provider arise, with an increase in failure rate. Therefore, the authors in [28] developed a task-scheduling approach which preserves reliability, i.e., RATSA. The scheduling of tasks onto resources was mapped using the frog-leaping algorithm to minimize makespan. RATSA was implemented on Cloudsim and compared with baseline mechanisms. Results proved that RATSA minimized the failure rate of tasks by 40%. Cost-effective scheduling is the primary facet of task scheduling, but to tackle scheduling in a cloud computing model with multiple objectives, it is necessary to use a nature-inspired algorithm as it is an NP-hard problem. Therefore, the authors in [29] used seagull optimization to design their scheduler. The Cloudsim tool was used for simulation. The model was compared to ACO, GA, PSO, and WOA algorithms and SOATS was compared to CJS, MSDE, and FUGE approaches, and it was observed that SOATS optimizes cost and energy by 10% and 25%, respectively. In [25,26,27,28,29,30], the authors concentrated mainly on datacenter processing time, QoS parameters, and task processing time, and only one author in [28] discussed failure rate as all the existing task-scheduling algorithms discussed various parameters. But many of the authors did not tackle the relationship between fault tolerance and trust-based parameters. Therefore, we reached a logical conclusion that we need to focus on tackling fault-tolerance and trust-based parameters.

From Table 1, we can observe that many authors used various nature-inspired algorithms and addressed various parameters, i.e., makespan, cost, execution time, and throughput, but a very limited number of authors discussed fault-tolerance and SLA-based trust-based parameters in the scheduling process in cloud computing. Therefore, we chose these parameters as evaluation criteria in the scheduling process. In this research work, we introduced a fault-tolerant trust-aware scheduling mechanism by capturing priorities of tasks and VMs carefully at different levels and addressed parameter failure rate, success rate, availability, and turnaround efficiency. This scheduling model was developed using Harris hawks optimization.

## 3. Fault-Tolerant Trust-Based Task Scheduling

### 3.1. FTTATS Problem Definition and System Architecture

**Figure 1:**
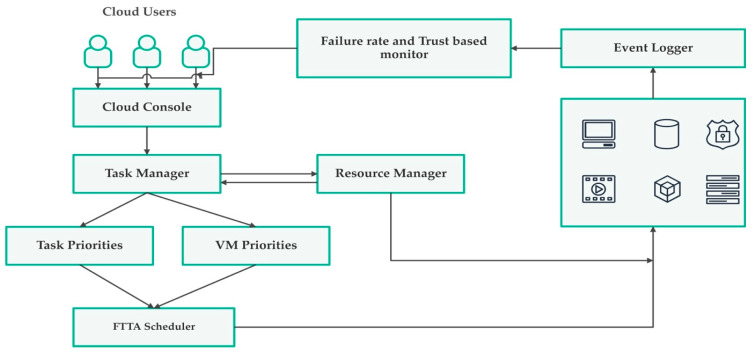
Proposed System Architecture.

In this subsection, we clearly formulated problem definition by considering the number of tasks as $t^{k}=\{t^{1},t^{2},t^{3},\ldots .t^{k}\}$, the number of VMs as ${vm}^{n}=\{{vm}^{1},{vm}^{2},{vm}^{3},\ldots {vm}^{n}\}$, the number of physical hosts as $h^{p}=\{h^{1},\;h^{2},\;h^{3},\ldots \;h^{p}\}$, the number of datacenters as ${dc}^{q}=\{{dc}^{1},\;{dc}^{2},\;{dc}^{3}\ldots \;{dc}^{q}\}$. Therefore, we formulated definition by assuming that these $t^{k}$ tasks are to be scheduled on to $vm^{n}$s which are placed in $h^{p}$ physical hosts. They reside in $dc^{q}$ datacenters by tackling success rate, failure rate, turnaround efficiency, and availability. Figure 1 below represents system architecture. Initially, various cloud users submit requests to the cloud interface. These requests are submitted to the task manager. After the submission of tasks, priority of tasks is evaluated. Priorities are carefully evaluated based on the size of task and the respective processing capacity of a VM to which it needs to be scheduled. After calculation of these priorities at the first level, VM priorities are calculated using electricity price at which that VM is located. These priorities need to be fed to the FTTA scheduler, which checks the priorities of both VMs and map high-prioritized tasks with high-prioritized VMs, i.e., VM residing in the low electricity cost region. While scheduling tasks to precise VMs, the task manager interacts with the resource manager in order to track resource utilization and updates resource status to the scheduler for every task assignment. In this research work, for evaluating and analyzing SLA-based trust parameters and failure rate, we added an event logger and captured the feedback to evaluate fault tolerance and trust parameters. Notations used in mathematical modelling and in the proposed system architecture are presented in Table 2 below.

### 3.2. FTTATS Mathematical Modelling

In this subsection, we precisely formulated mathematical modelling for our FTTATS scheduler. Initially, we identified and evaluated priorities of tasks to determine present workload on each virtual instance. Using Equation (1) below, present workload on all virtual instances was calculated.

(1) $$ld^{vm^{n}}=\sum ld^{n},$$

where $l{d^{vm}}^{n}$ is workload on all n VMs, $ld^{n}$ indicates load on each VM. All these VMs reside in physical hosts. Workload on all p physical hosts was evaluated using Equation (2) below.

(2) $$ld^{h^{p}}=\frac{ld^{vm^{n}}}{\sum h^{p}},$$

where $ld^{h^{p}}$ is workload on all physical hosts. For the calculation of priorities, one of the important steps is to identify the processing capacity of vm, the length of a task. The processing capacity of VM was evaluated using Equation (3).

(3) $$po^{vm}=po^{no}*po^{MIPS},$$

Initiallym we calculated the processing capacity of a single VM. Then, the entire processing capacity of all VMs was evaluated using Equation (4).

(4) $$to_{po}^{vm}=\sum po^{vm^{j}}.$$

After calculating the processing capacity of a VM, the length of a task was identified using Equation (5).

(5) $$t_{len}^{k}=t_{MIPS}^{k}*t_{po}^{k}.$$

The processing capacity of VMs and task length were evaluated using Equations (4) and (5) respectively. Priorities of all heterogeneous tasks from various users were evaluated using Equation (6) below.

(6) $$t_{pri}^{k}=\frac{t_{len}^{k}}{po^{vm^{j}}}.$$

In this work, after calculating priorities of tasks, VM priorities were calculated using electricity cost captured at various datacenters considered in our work. They were calculated using Equation (7) below.

(7) $$vm_{pri}^{j}=\frac{ecost_{high}}{ecost^{dc^{q}}}.$$

From Equations (6) and (7), we calculated priorities of tasks, VMs and highly prioritized tasks based on VM processing capacity. Task lengths and tasks were mapped according to priorities calculated using Equations (6) and (7), maping a task with the highest priority and mapping onto a VM with a high priority, i.e., with low electricity cost. If a VM with high priority is not available for high priority, then that task is mapped to the next prioritized VM. In our work, we included a deadline-aware constraint through which we precisely scheduled a task on a corresponding VM. Other tasks are not allowed execution on that VM until the pending task completes its execution, and deadline constraint in our work is indicated as ${d^{t}}^{k}$. The primary objective of any task scheduler is to minimize makespan. In our research, we identified that in order to calculate makespan, it is necessary to calculate the execution time of thetask on a corresponding VM. Therefore, we calculated execution time of a task on a VM using Equation (8) below.

(8) $$exe^{t^{k}}=\frac{exe^{t}}{po^{vm}}.$$

After the calculation of execution time in Equation (8), we identified the finish time of the task using Equation (9). For task $t^{k},$ when it enters the execution queue, it may immediately receive a VM, or it should wait for the other task to finish its execution. Therefore, the finish time of a task plays a key role in this aspect, and it is mentioned in Equation (9).

(9) $$fin^{time^{k}}=\sum vm^{n}+exe^{t^{k}}.$$

After identifying the finishing time for a task, we need to emphasize that finishing time should be shorter than the deadline of that corresponding task, and it is evaluated using Equation (10).

(10) $$fin^{time^{k}}\leq d^{t^{k}},$$

Then, we evaluated various parameters. Primarily, we evaluated the makespan. It was measured based on the time of execution of a task on a chosen VM. If the execution time increases and makespan increases, scheduler performance degrades. For effective scheduling of tasks, makespan needs to be minimized. It is calculated using Equations (11) and (12).

(11) $$m^{k}=max\left(fin^{time_{vm^{n}}}\right),$$

(12) $$minfin^{time}\left(t^{k}vm^{n}\right)=\sum_{r=1}^{k}\sum_{s=1}^{n}\delta_{rs}fin^{time}\left(t^{k}vm^{n}\right).$$

From Equation (12), we can observe that δ is assigned to a value of zero or one based on task assignment to a VM. If task $t^{k}$ is assigned to VM $vm^{n},$ then the value is set to one, otherwise it is to be considered zero. After calculating the makespan identifying the rate of failures of tasks, our aim is to develop a fault-tolerant trust-based scheduler. Failure rate can be defined as a ratio of the number of failed tasks in the proposed approach to the number of failed tasks with existing approaches. It is calculated using Equation (13) below.

(13) $$RF=\frac{\sum_{0}^{b}No.of\;failures\;for\;FTTA}{\sum_{0}^{b\;}No.of\;failures\;for\;existing\;approaches\;}.$$

After identifying the rate of failures from Equation (13), we focused on identifying SLA-based trust parameters as the failure rate is related with trust, because whenever the rate of failures decreases, the trust of cloud provider improves because of the improvement in SLA-based trust parameter availability of VM, success rate of a VM, and turnaround efficiency. Availability of VM is an important parameter related to trust in a cloud provider, because if a user requests a resource and it is provisioned seamlessly to the user without any problems, then the availability of a resource chosen by the user is high in the respective cloud provider. Automatically, the trust in the cloud provider increases with respect to availability. Availability of VM is defined as the ratio of the number of tasks accepted by VM to the total number of tasks. Equation (14) below indicates the availability of VMs.

(14) $$a\left(vm^{n}\right)=\frac{a^{k}}{t^{k}}.$$

After deduction of the availability of a VM, another trust-based parameter is the success rate of a VM. It is calculated as the ratio of the number of successful requests executed to submitted requests on a corresponding VM according to SLA. It is calculated using Equation (15).

(15) $$SR\left(vm^{n}\right)=\frac{suc^{t^{k}}}{sub^{t^{k}}}.$$

Another important parameter that plays a key role in maintaining trust in a cloud provider is turnaround efficiency. It is defined as the ratio of estimated turnaround time of a task which is mentioned in SLA to the actual turnaround time for executing a task on a respective virtual resource. It is shown here in Equation (16).

(16) $$tt\left(vm^{n}\right)=\frac{es^{t^{k}}}{act^{t^{k}}}\;.$$

Then, after evaluating availability, success rate, and turnaround efficiency of a VM, we calculated trust in a cloud provider and it is calculated using Equation (17) below.

(17) $$tr^{CP}=S1*a\left(vm^{n}\right)+S2*SR\left(vm^{n}\right)+S3*tt\left(vm^{n}\right),$$

In Equation (17) above, $S=\left\{S1,S2,S3\right\}$ are positive weights, and based on these values, trust value is evaluated. These values are taken from [39]. These are positive weights, and the range of values lies in-between zero and one, and these values are calculated using the co-variance technique. From [39], we assumed the weights for availability as S1=0.5, success rate as S2=0.2, turnaround efficiency as S3=0.1. From Equation (17), trust in the cloud provider was calculated using the above-mentioned weights.

### 3.3. Fitness Function for FTTATS

After mathematical modelling of FTTATS, we carefully model the fitness function for FTTATS to optimize the parameters addressed in our research. It is calculated using the following Equation (18).

(18) $$f\left(x\right)=\alpha_{1}*m^{k}+\alpha_{2}*RF+\alpha_{3}*a\left(vm^{n}\right)+\alpha_{4}*SR\left(vm^{n}\right)+\alpha_{5}*tt\left(vm^{n}\right),$$

(19) $$\alpha_{1}+\alpha_{2}+\alpha_{3}+\alpha_{4}+\alpha_{5}=1.$$

From Equations (18) and (19), fitness function is calculated for the optimization of parameter makespan, rate of failures, success rate, availability, turnaround efficiency to gain trust in the cloud provider and to improve fault tolerance. In the next subsection, we model a fault-tolerant trust-aware task scheduler (FTTATS) using Harris hawks optimization in a detailed manner.

### 3.4. Fault-Tolerant Trust-Aware Task Scheduler (FTTATS) Using Harris Hawks Optimization

This subsection briefly discusses the methodology used in the FTTATS schedulerl i.e., Harris hawks optimization, which is a nature-inspired algorithm based on the catching and hunting behaviour of hawks for rabbits as presented in [40]. This algorithm consists of different phases to catch and hunt prey, i.e., exploration, exploitation, and transition. Initially, in this algorithm, hawks keenly wait to catch their prey based on its location, i.e., prey lies in a group or it is away from group. Therefore, to identify the position of prey, i.e., rabbit, in this case, the data is calculated using Equation (20) below.

(20) $$X\left(T+1\right)=\left\{\begin{array}{c} Q\geq 0.5\;\;\;\;\;\;\;X^{RAND}\left(T\right)-P_{1}\left|X^{RAND}\left(T\right)-2P_{2}X\left(T\right)\right|\\ Q<0.5\;\;\;\left(\;X^{RAB}\left(T\right)-X^{m}\left(T\right)\right)-P_{3}\left(LB+P_{4}\left(UB-LB\right)\right) \end{array}\right.,$$

where $X\left(T+1\right)$ is hawk position in the next iteration, $X^{RAB}\left(T\right)$ is the current position of the rabbit, $P_{1}$, $P_{2}$, $P_{3}$, $P_{4}$ are current position vectors of hawks. Q is a random number which lies inside the range of 0,1. UB and LB are upper and lower bounds of variables, $X^{RAND}\left(T\right)$ is a randomly selected hawk bird from a population, $X^{m}\left(T\right)$ is the average position of a hawk bird among the population.

The average hawk position is calculated using the following Equation (21):

(21) $$X^{m}\left(T\right)=\frac{1}{U}\sum_{c=1}^{U}X_{c}^{m}\left(T\right).$$

After the evaluation of a hawk bird’s position, the energy of prey, i.e., rabbit’s energy, is evaluated using Equation (22) below.

(22) $$EN=2EN_{0}\left(1-\frac{T}{R}\right),$$

where R is the maximum number of iterations, EN is the required escaping energy for a rabbit, i.e., prey, $EN_{0}$ is the initial energy required for the prey to escape from a hawk bird. The range of energy for prey lies in-between [–1,1]. When the initial energy of prey, i.e., $EN_{0},$ decreases from 0 to −1, then it can be easily hunted by a hawk bird, and when the initial energy of prey, i.e., $EN_{0},$ increases from 0 to 1, then the chances of escaping from a hawk bird increases before the exploitation phase. The transition of prey from exploration to exploitation mainly depends on two parameters, (1) required energy for prey to escape from hunting and (2) probability of chance to escape from a hawk bird. In our research, the probability of escaping from a hawk bird is denoted as P. In the first case, if P<0.5, there is an escaping chance for prey from a hawk bird, and in the second case, if P≥0.5, then there is no chance of escaping for prey from a hawk bird. Generally, in Harris hawks optimization, exploitation is categorized into several types: soft besiege, hard besiege, soft besiege with incremental steps, and hard besiege with incremental steps [40]. Soft besiege occurs based on two conditions: if P≥0.5 and |EN|≥ 0.5, and it is evaluated using = Equations (23) and (24).

(23) $$X\left(T+1\right)=\psi X\left(T\right)-EN|WX^{RAB}\left(T\right)-X\left(T\right)|,$$

(24) $$\psi X\left(T\right)=X^{RAB}\left(T\right)-X\left(T\right),$$

$\Psi X\left(T\right)$ is the difference between the position vector of the rabbit and the current iteration at T.

After the calculation of soft besiege, hard besiege occurs based on two conditions: if P≥0.5 and |EN|≤ 0.5, and it is evaluated using Equation (25).

(25) $$X\left(T+1\right)=X^{RAB}\left(T\right)-EN|\psi X\left(T\right)|.$$

After the calculation of hard besiege, there are other categories of besieges: for example, soft besiege with incremental steps, and it is based on previous movements of the hawk bird. It is calculated as follows, using Equation (26):

(26) $$Y=X^{RAB}\left(T\right)-EN|WX^{RAB}\left(T\right)-X\left(T\right).$$

In the above besiege, hawk movement is based on the previous step of the hawk bird, and if the steps of the hawk bird are not constructive, then the hawk bird abruptly encircles the prey. It is evaluated using Equation (27).

(27) $$Z=Y+b*LF\left(di\right),$$

where di is the dimension of the problem, and LF is the levy flight function.

(28) $$LF\left(Y\right)=0.01*\frac{\lambda *\kappa }{|\zeta {|}^{\frac{1}{\eta }}},$$

(29) $$\kappa ={\left(\frac{\kappa \left(1+\eta \right)*\sin\left(\frac{\pi \eta }{2}\right)}{\kappa \frac{\left(1+\eta \right)}{2}*\eta *2^{\frac{\eta -1}{2}}}\right)}^{1/\eta },$$

where λ, ζ are random values that lay in-between 0, 1. The η value is set to 1.5 as per [40].

For soft besiege with constructive or incremental steps, updation is performed by using the following Equation (30):

(30) $$X\left(T+1\right)=\left\{\begin{array}{c} Y\;\;\;\;\;\;\;f\left(Y\right)<f\left(X\left(T\right)\right)\\ Z\;\;\;\;\;\;\;f\left(Z\right)<f\left(X\left(T\right)\right) \end{array}\right..$$

In the above equation, Y, Z are calculated in Equations (26) and (27) respectively. For hard besiege with constructive or incremental steps, updation is performed by using the following Equation (31):

(31) $$X\left(T+1\right)=\left\{\begin{array}{c} Y\;\;\;\;\;\;\;f\left(Y\right)<f\left(X\left(T\right)\right)\\ Z\;\;\;\;\;\;\;f\left(Z\right)<f\left(X\left(T\right)\right) \end{array}\right..$$

In the above equations, Y, Z are calculated in Equations (32) and (27) respectively.

(32) $$Y=X^{rab}\left(T\right)-EN|WX^{rab}\left(T\right)-X^{m}\left(T\right).$$

$X^{m}\left(T\right)$ is calculated from Equation (21).

### 3.5. Proposed FTTA Task-Scheduling Algorithm

The proposed fault-tolerant trust-aware task-scheduling algorithm using Harris hawks optimization in cloud computing is presented below as Algorithm 1.
**Algorithm 1.** Fault-tolerant trust-aware task scheduling algorithm using Harris hawks optimization.**Input:**$t^{k}=\{t^{1},t^{2},t^{3},\ldots .t^{k}\}$$,\;{vm}^{n}=\{{vm}^{1},{vm}^{2},{vm}^{3},\ldots {vm}^{n}\}$$,\;h^{p}=\{h^{1},\;h^{2},\;h^{3},\ldots \;h^{p}\}$, ${\;dc}^{q}=\{{dc}^{1},\;{dc}^{2},\;{dc}^{3}\ldots \;{dc}^{q}\}$.
**Output:** Efficient generation of schedules for tasks by mapping them to precise VMs while minimizing $m^{k}$, RF, and improving $a{\left(VM\right)}^{n}$, $SR{\left(vm\right)}^{n}$, $tt{\left(vm\right)}^{n}$, $tr^{CP}$.
  Start

  Initialization of Hawk birds population in a random manner.

  Initialization of fitness function.

  Calculation of task priorities by Equation (6).

  Calculation of VM priorities by Equation (7).

  Calculate fitness function by Equation (18).

  if|EN≥1| then

  Update position vectors using Equation (20).

  else

  if|EN<1|

  It is in exploitation.

  $if\left(P\geq 0.5\right)\&\&(|EN|\geq 0.5)$
 then

  Soft besiege begins, position vectors updation by Equation (23).

  $elseif\left(P\geq 0.5\right)\&\&(\left|EN\right|<0.5)$
 then

  Hard besiege begins, position vectors updation by Equation (25).

  $elseif\left(P<0.5\right)\&\&(\left|EN\right|\geq 0.5)$  then

  Soft besiege by constructive steps begin, position vectors updation by Equation (30).

  $elseif\left(P<0.5\right)\&\&(\left|EN\right|<0.5)$  then

  Hard besiege by constructive steps begin, position vectors updation by Equation (31).

  Identify best mapped tasks to VMs using above Hawks and calculate $m^{k}(x),\;RF(x)$$,\;a{\left(VM\right)}^{n}$$\left(x),\;SR{\left(vm\right)}^{n}(x\right)$$,\;tt{\left(vm\right)}^{n}(x)$.

  if current trust is increased then add current trust value to existing trust value of cloud provider.

  else

  Trust value of cloud provider exponentially decreases

  end if

  end if

  end if

  Repeat process until all iterations completed.
  End

**Figure 2:**
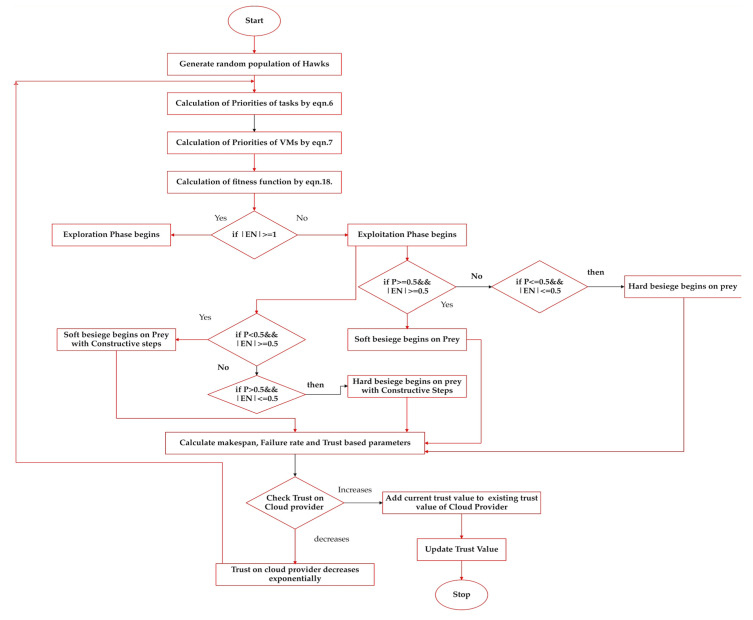
Flow of Proposed FTTA task scheduler.

Figure 2 above represents the flow of the proposed FTTA task scheduler in which a random hawk population is initially generated. In the next step, priorities of tasks and VMs are calculated using Equations (6) and (7). After calculating priorities, fitness function is evaluated by Equation (18). There are two phases in Harris hawks optimization, i.e., exploration and exploitation, as mentioned in [40]. In the exploration phase, if |EN≥1|, then the hawk bird searches for prey until it is discovered, and position vectors can be updated using Equation (20). Otherwise, the exploitation phase begins to catch the prey, but in this phase, there are other two cases, i.e., soft besiege occurs when the probability of escaping for the prey ≥ 0.5, the energy of prey is ≥ 0.5, and if the probability of escaping for the prey ≥ 0.5, the energy for the prey is ≤ 0.5, then hard besiege occurs. When soft besiege occurs, the position vectors are updated using Equation (23), and for hard besiege, position vectors are updated using Equation (25). In this exploitation phase, there is another case when the probability of escaping for the prey is less than 0.5, energy ≥ to 0.5; then, soft besiege occurs in prey with constructive steps, and if not, then hard besiege in prey with constructive steps is applied. For soft besiege with constructive steps, position vectors are updated using Equation (30), and for hard besiege with constructive steps, position vectors are updated using Equation (31). After these steps, the values for makespan are identified, as well as the rate of failures, availability, the success rate of VMs, the turnaround efficiency through which trust in the cloud provider is evaluated. Thereafter, trust in the cloud provider is identified, and if it is increased over the existing trust value, it is updated as the current trust value, and if it is not increased, it isexponentially decreased. The same process is repeated until all iterations are completed in the FTTA task-scheduling mechanism.

## 4. Simulation and Results

This section mainly represents extensive simulations carried out for FTTATS using random generated workload, statistical dispersion of tasks using various fabricated datasets by different distributions and realtime worklogs from [41,42]. This extensive set of experiments conducted using the Cloudsim [43] simulator to evaluate the performance of the FTTATS scheduler. Initially, to evaluate all parameters, we used various statistical data distributions by fabricating the datasets, i.e., represented as D01, D02, D03, D04, i.e., uniform, normal, left skewed, and right skewed, respectively. After the calculation of makespan with these, we chose real-time worklogs from [41,42], and they were represented as D05 and D06 throughout the research. We chose these dataset for fabrication in this simulation because generally, many existing authors used random generated workloads, but using a random generated workload for this type of scheduling problem does not provide precise schedules. Therefore, we fabricated different distributions of datasets, D01–D04, and to check the efficacy of the approach, we chose real-time worklogs from the HPC2N computing cluster and NASA worklogs. This section consists of various subsections including configuration settings required for simulation, calculation of makespan, rate of failures, availability, success rate, and turnaround efficiency of VMs. A precise discussion on the generated results and analysis is represented in another subsection.

### 4.1. Simulation Setup and Configuration Settings

This subsection clearly represents simulation setup and standard configuration settings used in our simulation. To evaluate the proposed FTTATS, we compared the proposed FTTATS with the existing state-of-the-art approaches, ACO, GA, and PSO. We used configuration settings required for simulation captured from [42]. Table 3 below represents configuration settings used in the simulation for the proposed FTTATS.

### 4.2. Evaluation of Makespan

**Figure 3:**
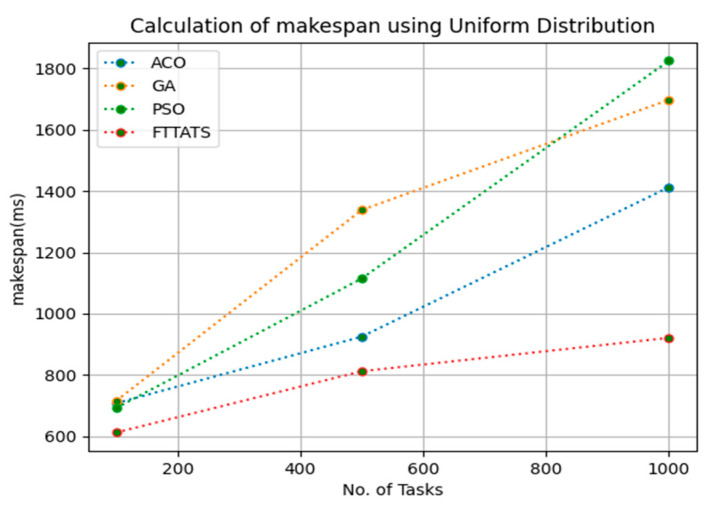
Makespan calculation by D01.

**Figure 4:**
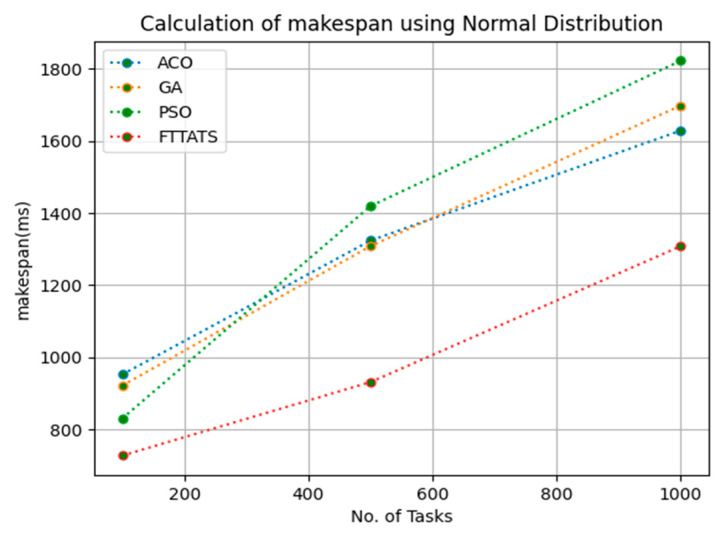
Makespan calculation by D02.

**Figure 5:**
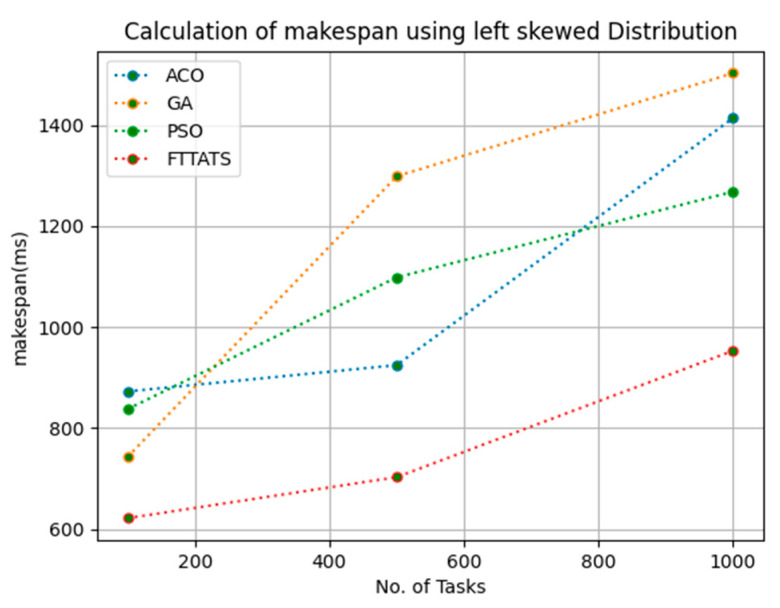
Makespan calculation by D03.

**Figure 6:**
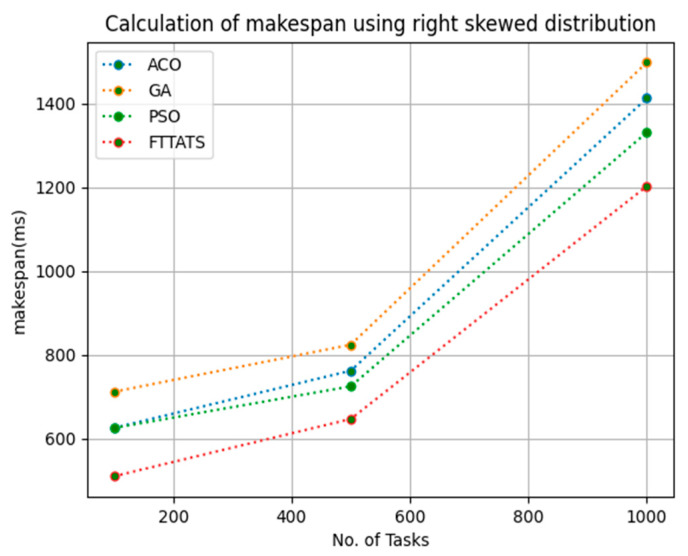
Makespan calculation by D04.

**Figure 7:**
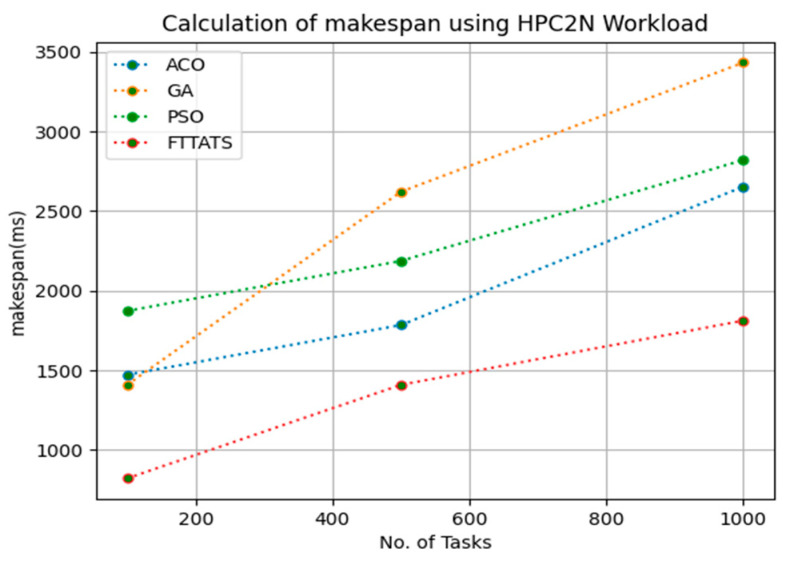
Makespan calculation by D05.

**Figure 8:**
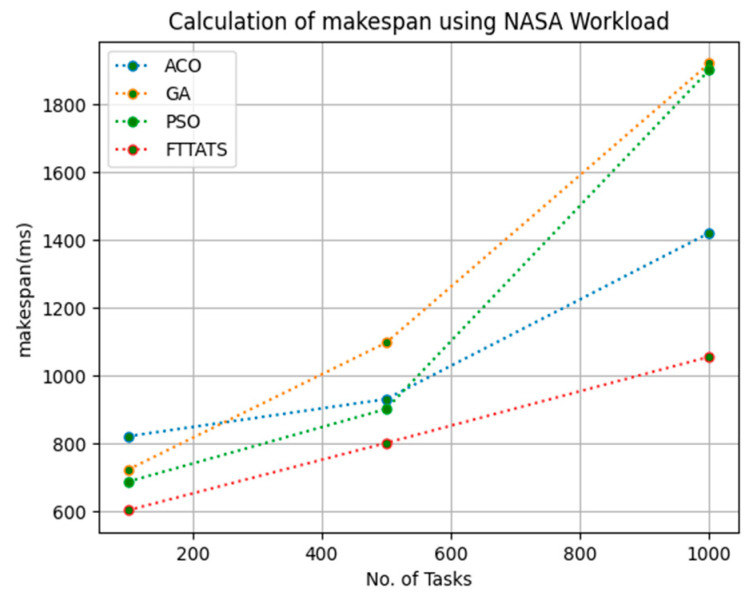
Makespan calculation by D06.

In this subsection, the makespan of all tasks submitted to the scheduler is calculated. Here, at first, we calculated the makespan as it is an important perspective in the scheduling process of the cloud paradigm. The effectiveness of the scheduling process in the cloud model depends on the minimization of the makespan. Therefore, we chose this parameter as the primary concern for our research; to showcase the efficacy of the proposed FTTATS, it was compared against the existing ACO, GA, and PSO algorithms. Table 4 below and Figure 3, Figure 4, Figure 5, Figure 6, Figure 7 and Figure 8 show the generated makespan for different distributions of data, i.e., D01, D02, D03, D04, and real-time worklogs, i.e., D05 and D06. From the generated makespan, it is evident that the proposed FTTATS outperformed the existing approaches in regard to makespan. 

### 4.3. Evaluation of Rate of Failures

**Figure 9:**
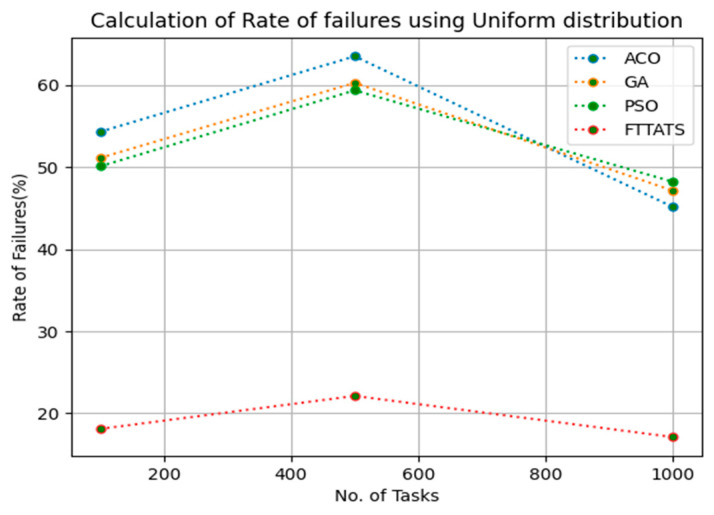
Rate of Failures calculation by D01.

**Figure 10:**
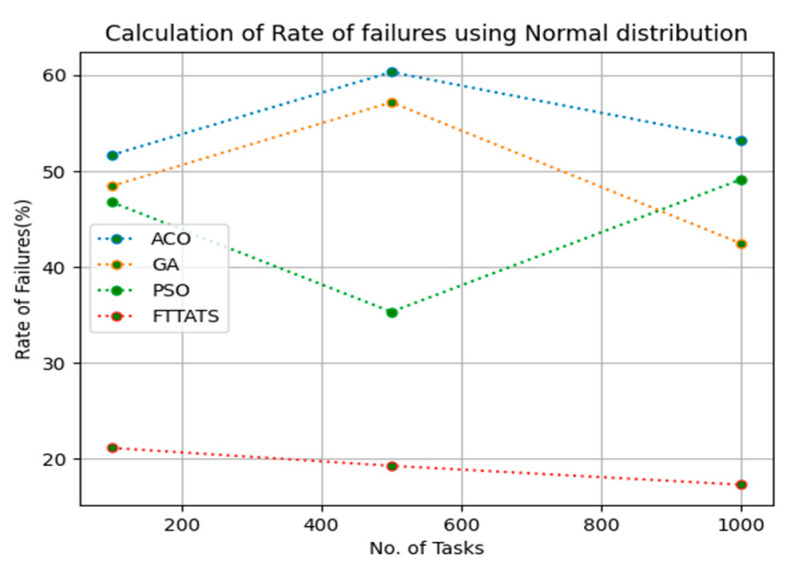
Rate of Failures calculation by D02.

**Figure 11:**
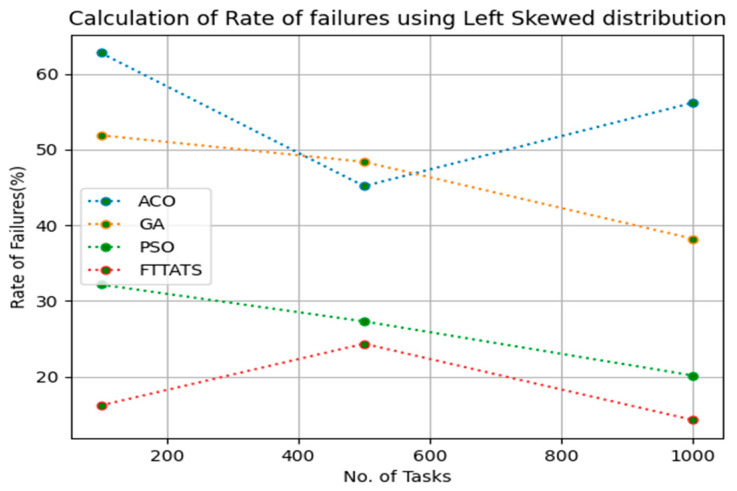
Rate of Failures calculation by D03.

**Figure 12:**
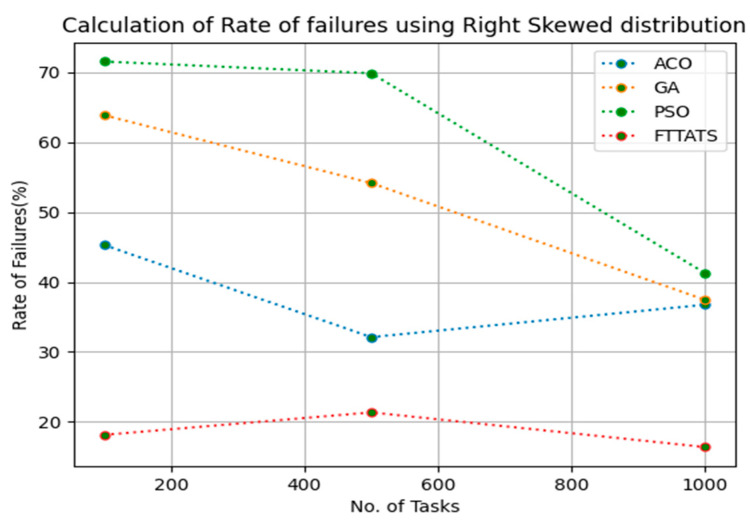
Rate of Failures calculation by D04.

**Figure 13:**
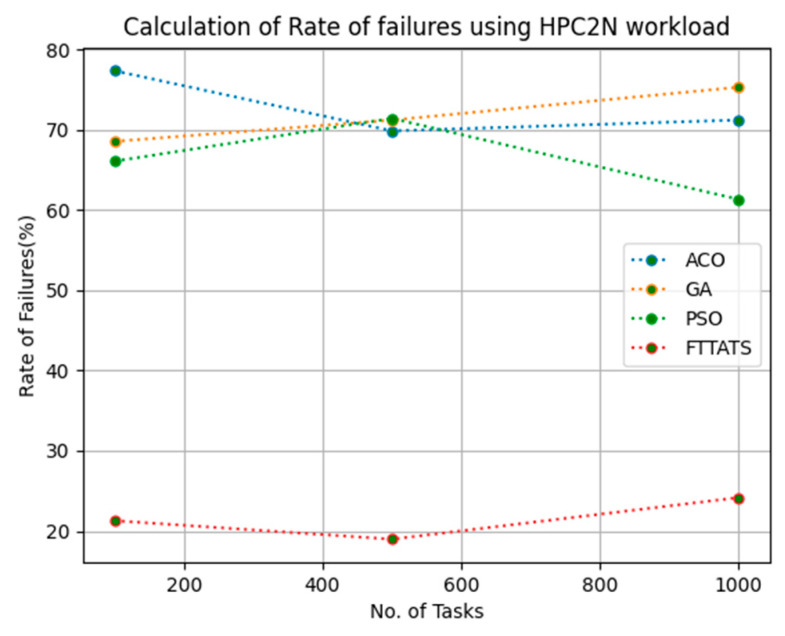
Rate of Failures calculation by D05.

**Figure 14:**
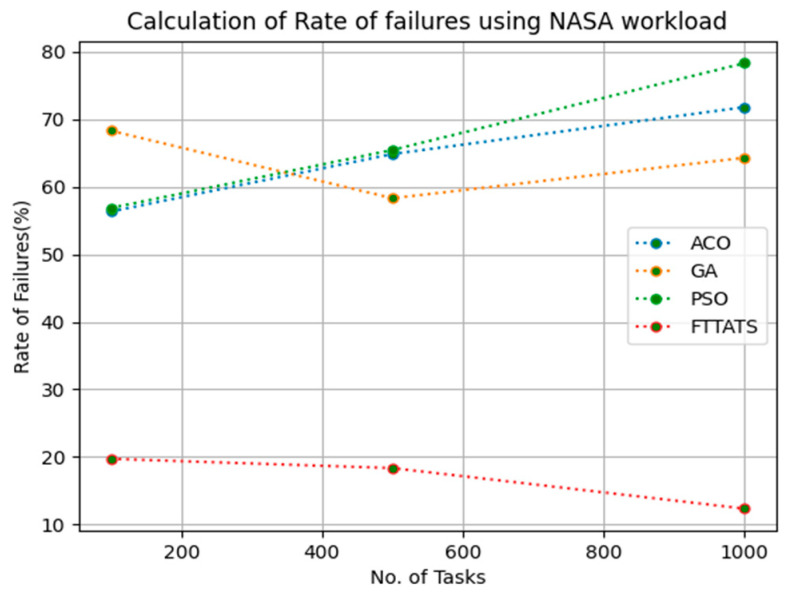
Rate of Failures calculation by D06.

In this subsection, after calculating the makespan, our next objective is to calculate the rate of failures, as in this research our model needs to generate schedules precisely while minimizing the rate of failures. We chose rate of failures as a parameter in this research because a single point of failures frequently occurs in the cloud environment, and it is the responsibility of the cloud provider to choose the backup model to sustain applications in the cloud paradigm and to gain the trust over their cloud environment from the customers. Therefore, the rate of failures is calculated in this research using Equation (13). Initially, fabricated datasets with different distributions are used, i.e., D01, D02, D03, and D04, and after that, D05 and D06 are used to calculate the rate of failures. The proposed FTTATS was compared to the existing ACO, GA, and PSO. Table 5 below and Figure 9, Figure 10, Figure 11, Figure 12, Figure 13 and Figure 14 show the generated rate of failures for different distributions of data, i.e., D01, D02, D03, D04, and real-time worklogs, i.e., D05 and D06. From the generated rate of failures, it is evident that the proposed FTTATS outperformed the existing approaches in regard to rate of failures.

### 4.4. Evaluation of Availability of VMs

**Figure 15:**
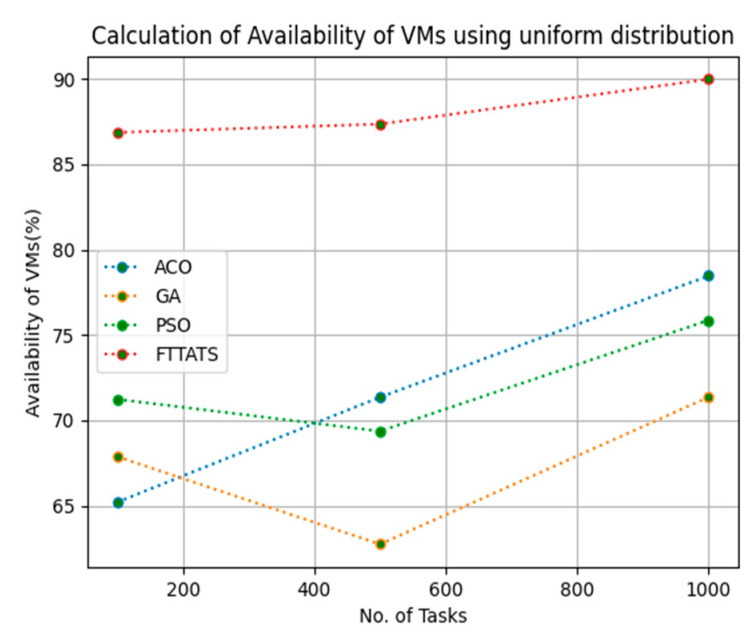
Availability of VMs calculation by D01.

**Figure 16:**
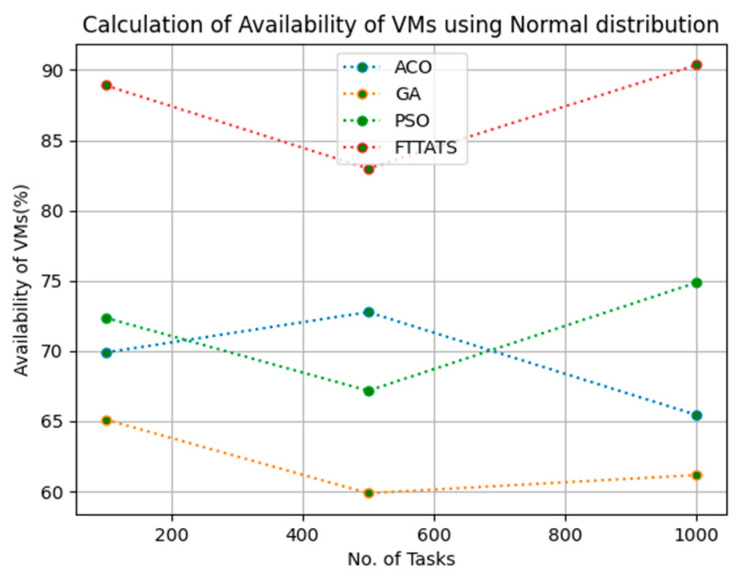
Availability of VMs calculation by D02.

**Figure 17:**
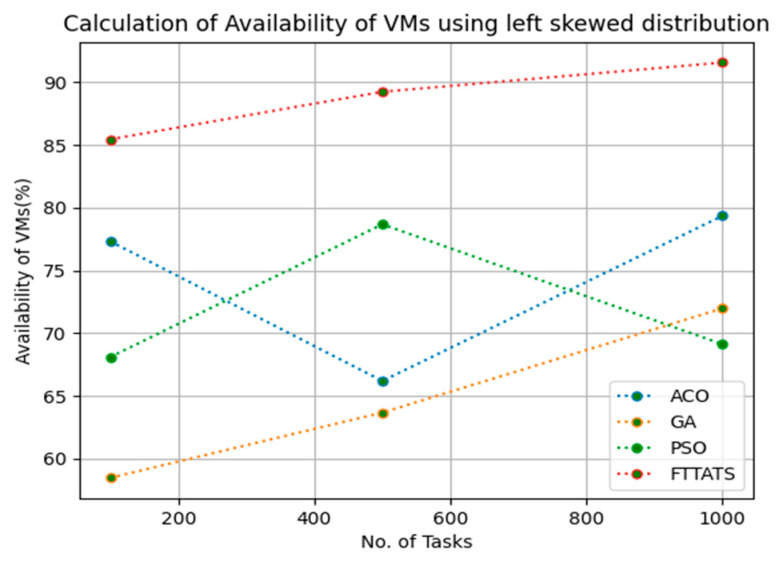
Availability of VMs calculation by D03.

**Figure 18:**
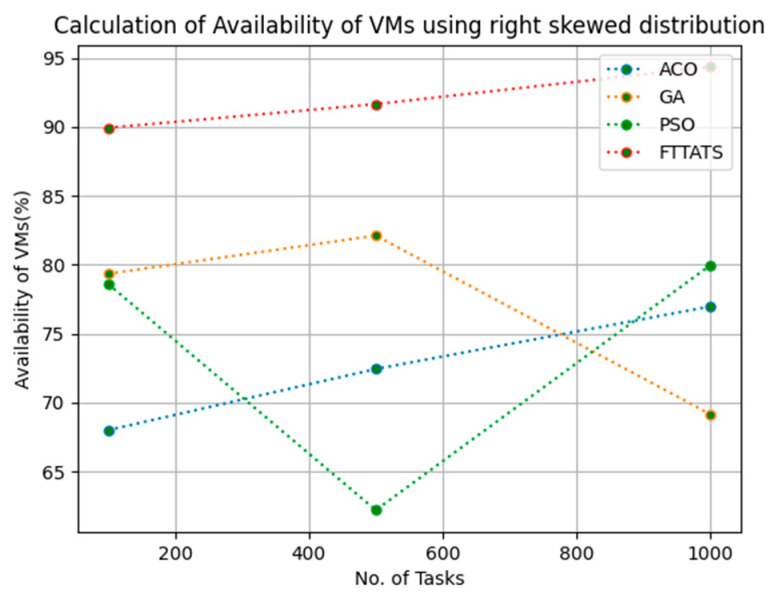
Availability of VMs calculation by D04.

**Figure 19:**
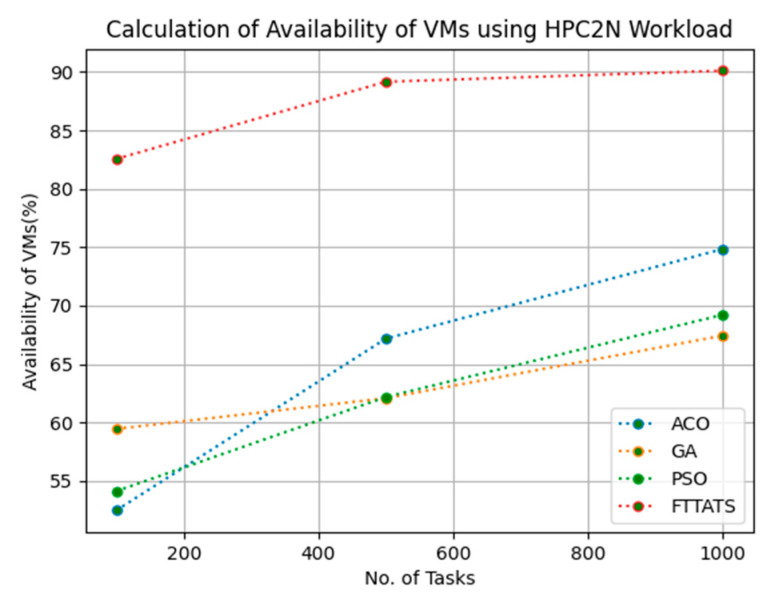
Availability of VMs calculation by D05.

**Figure 20:**
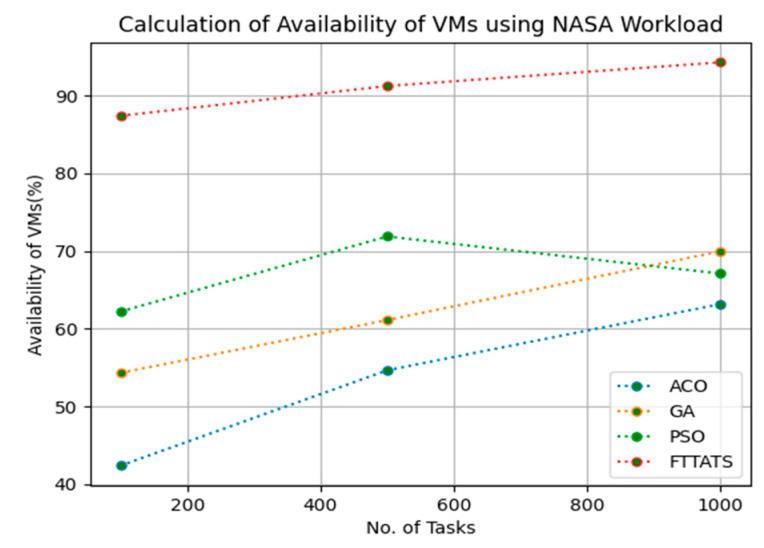
Availability of VMs calculation by D06.

In this subsection, we calculated our next parameter, i.e., availability of VMs. Availability plays a key role in the cloud paradigm as it is one of the factors to gain trust over the cloud provider. For this calculation, we used data distributions, i.e., D01, D02, D03, and D04, and real-time work logs, i.e., D05 and D06, as mentioned in the simulation setup. This simulation is needed to calculate availability. the proposed FTTATS was compared to state-of-the-art algorithms, i.e., ACO, PSO, and GA. Table 6 below and Figure 15, Figure 16, Figure 17, Figure 18, Figure 19 and Figure 20 show generated availability for different distributions of data, i.e., D01, D02, D03, and D04, and real-time worklogs, i.e., D05 and D06. From the generated availability, it is evident that the proposed FTTATS outperformed the existing approaches in regard to the availability of VMs.

### 4.5. Evaluation of Success Rate of VMs

**Figure 21:**
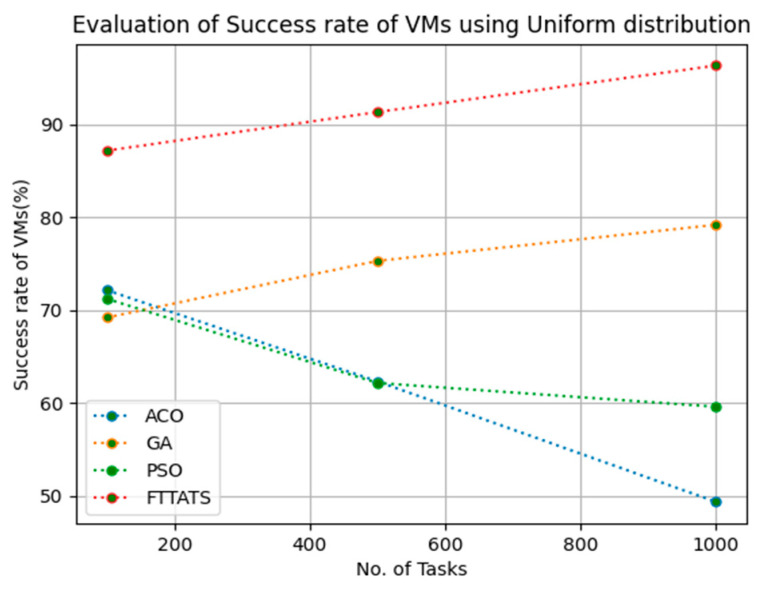
Success rate of VMs calculated by D01.

**Figure 22:**
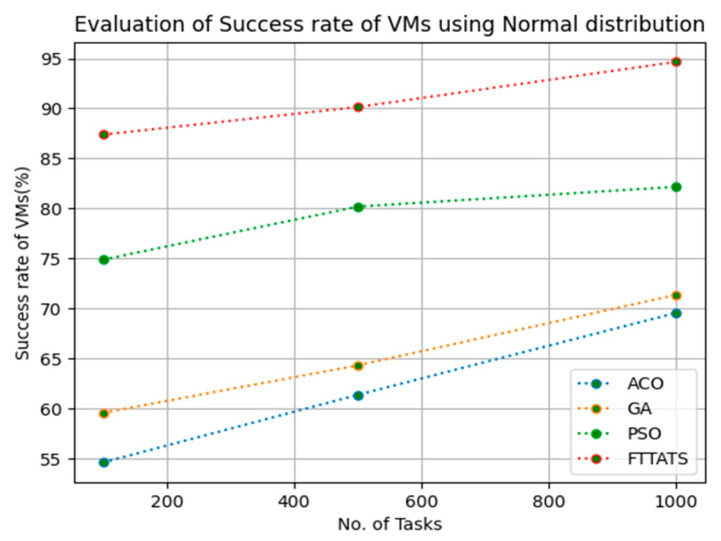
Success rate of VMs calculated by D02.

**Figure 23:**
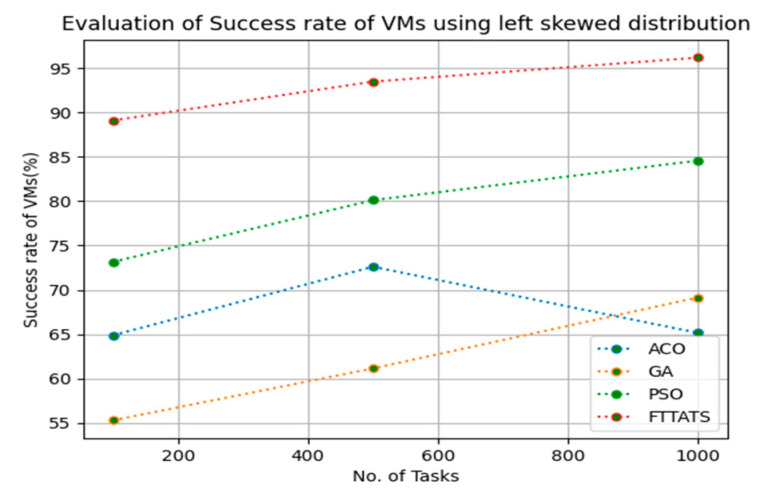
Success rate of VMs calculated by D03.

**Figure 24:**
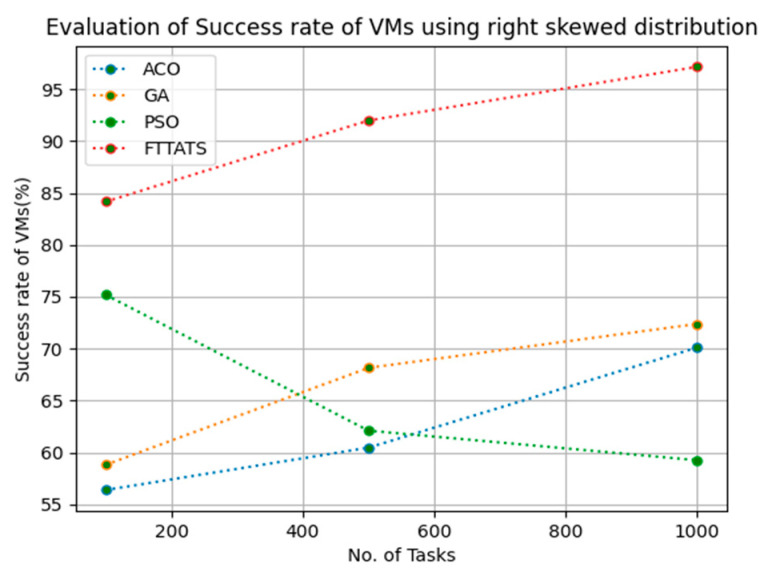
Success rate of VMs calculated by D04.

**Figure 25:**
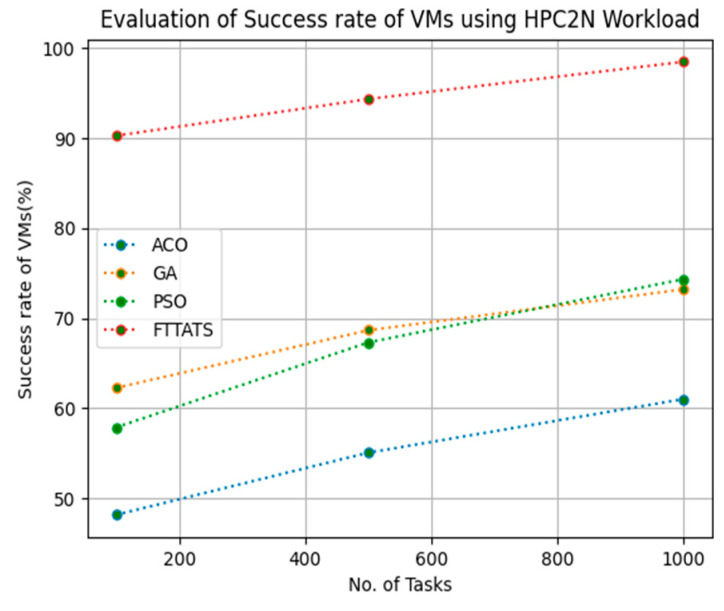
Success rate of VMs calculated by D05.

**Figure 26:**
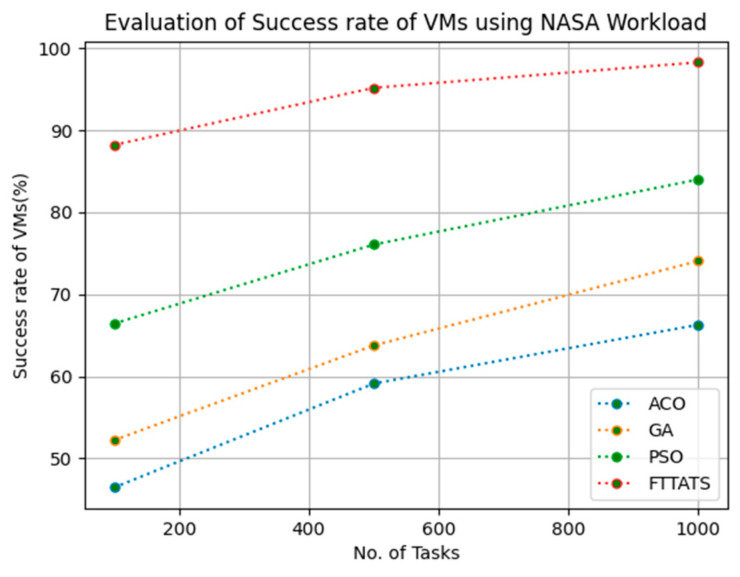
Success rate of VMs calculated by D06.

In this subsection, the success rate of VMs is evaluated by considering configuration settings mentioned in Table 3. We chose success rate of VM as a parameter because when a task is successfully executed on a VM, based on that, the success rate of a VM is evaluated, which is indirectly related to trust in the cloud provider. Therefore, to evaluate the success rate of VMs, we considered fabricated data distributions, i.e., D01, D02, D03, and D04, and real-time worklogs, i.e., D05 and D06. This simulation is used to calculate success rate. The proposed FTTATS was compared to state-of-the-art algorithms, i.e., ACO, PSO, and GA. Table 7 below and Figure 21, Figure 22, Figure 23, Figure 24, Figure 25 and Figure 26 show generated success rate for different distributions of data, i.e., D01, D02, D03, and D04, and real-time worklogs, i.e., D05 and D06. From the generated success rate, it is evident that the proposed FTTATS outperformed the existing approaches in regard to the success rate of VMs.

### 4.6. Evaluation of Turnaround Efficiency

**Figure 27:**
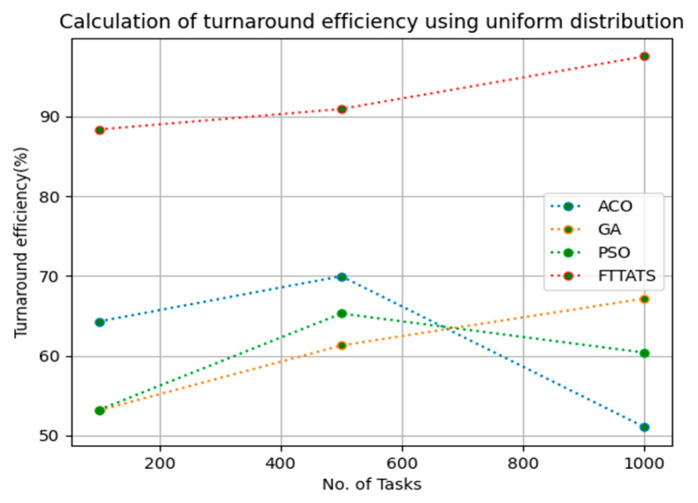
Turnaround efficiency of VMs calculated by D01.

**Figure 28:**
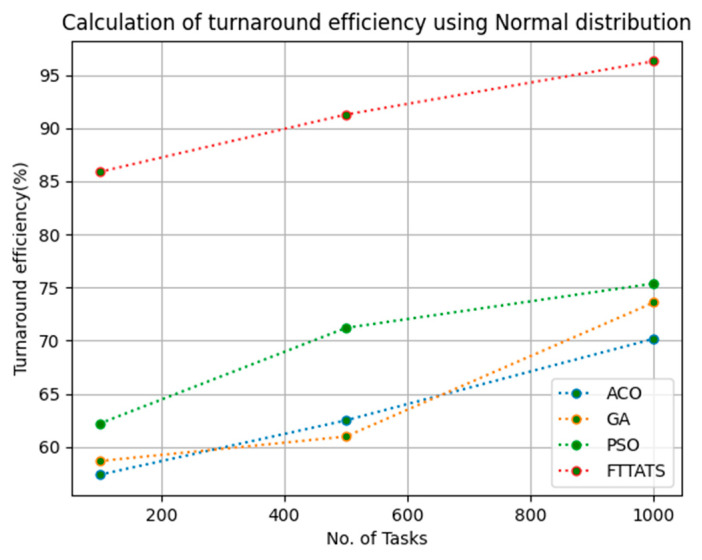
Turnaround efficiency of VMs calculated by D02.

**Figure 29:**
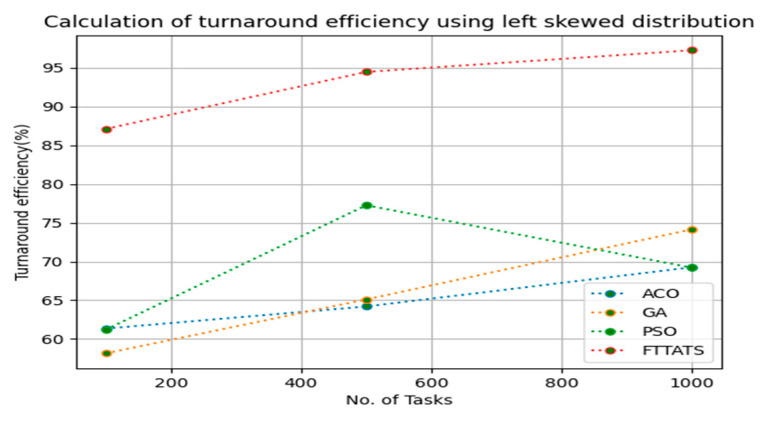
Turnaround efficiency of VMs calculated by D03.

**Figure 30:**
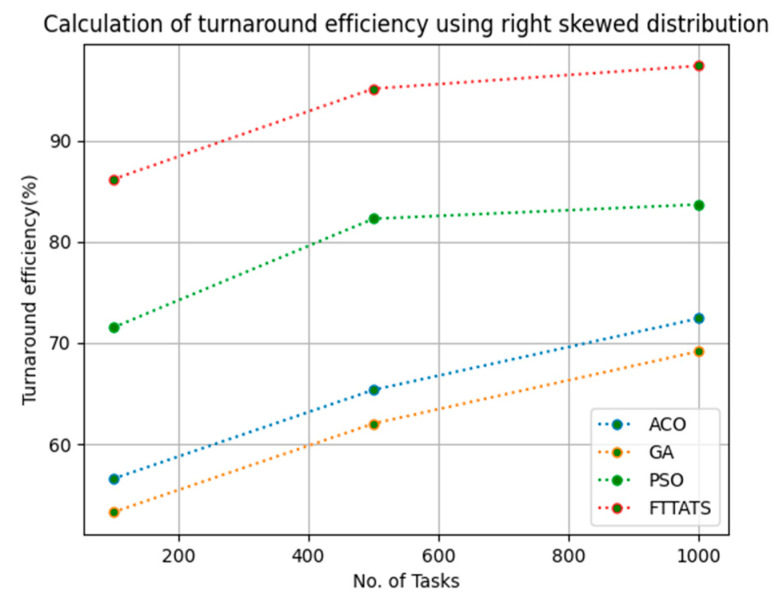
Turnaround efficiency of VMs calculated by D04.

**Figure 31:**
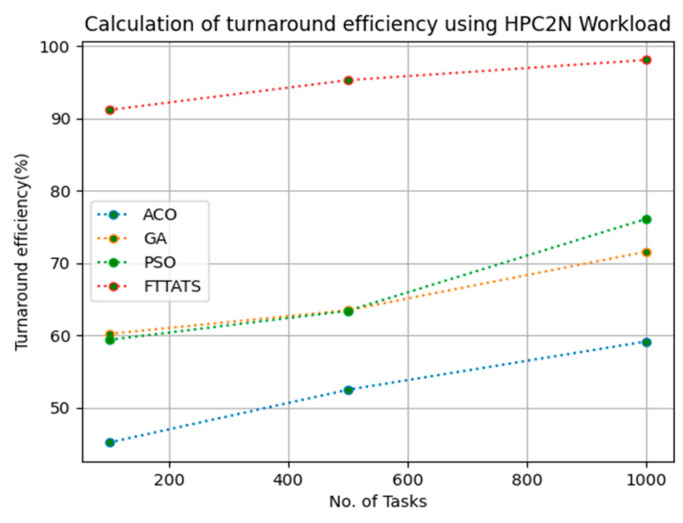
Turnaround efficiency of VMs calculated by D05.

**Figure 32:**
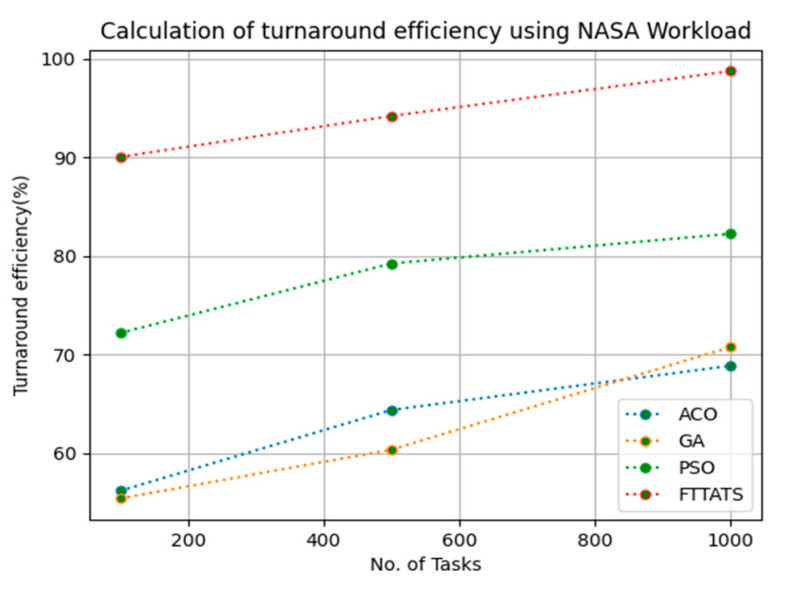
Turnaround efficiency of VMs calculated by D06.

In this subsection, turnaround efficiency is evaluated by considering configuration settings mentioned in Table 3. We chose turnaround efficiency as an evaluation parameter as it is related to the quality of service and time taken for a VM to take the submitted request of a user to respond to that request from the cloud provider. Therefore, to evaluate turnaround efficiency of VMs, we considered fabricated data distributions i.e., D01, D02, D03, and D04, and real-time worklogs, i.e., D05 and D06. This simulation is used to calculate turnaround efficiency. The proposed FTTATS was compared to state-of-the-art algorithms, i.e., ACO, PSO, and GA. Table 8 below and Figure 27, Figure 28, Figure 29, Figure 30, Figure 31 and Figure 32 show generated turnaround efficiency for different distributions of data, i.e., D01, D02, D03, and D04, and real-time worklogs, i.e., D05 and D06. From the generated turnaround efficiency, it is evident that the proposed FTTATS outperformed the existing approaches in regard to turnaround efficiency of VMs.

### 4.7. Analysis and Result Discussion

This subsection clearly presents analysis of results and discusses the ways in which the proposed FTTATS improves scheduling while addressing parameter makespan, rate of failures, success rate of VMs, availability, and turnaround efficiency. We conducted an extensive set of simulations on Cloudsim [43]. Initially, we fabricated datasets with different statistical distributions, and they are represented as D01, D02, D03, and D04, respectively. After evaluating FTTATS with statistical distributions, we used real-time worklogs, i.e., HPC2N [41] and NASA [42]. They are represented as D05, D06. The proposed FTTATS approach was evaluated in comparison to the existing state-of-the-art approaches, ACO, GA, and PSO. Table 9 represents the improvement of makespan for FTTATS over that of the existing approaches. Table 10 indicates improvement of rate of failures for FTTATS over that of the existing approaches. Table 11 indicates improvement of availability of VMs for FTTATS over that of the existing approaches. Table 12 indicates improvement of success rate of VMs for FTTATS over that of the existing approaches. Table 13 indicates improvement of turnaround efficiency of VMs for FTTATS over that of the existing approaches. From all these results, it was observed that the proposed FTTATS showed significant impact on generating schedules by minimizing makespan and rate of failures and improving SLA-based trust metrics mentioned above, leading to improvement of quality of service which, in turn, facilitates trust in the cloud provider. The main difference between other approaches and our proposed FTTATS is that the existing task schedulers are not considering priorities of tasks and VMs. Our proposed FTTATS improves rate of failures, makespan, availability, success rate and turnaround efficiency compared to those of the existing approaches, as mentioned in tables below.

## 5. Conclusions and Future Works

Task scheduling is the biggest challenge in cloud paradigm, as there is a wide variety of tasks generated from heterogeneous resources. These are to be scheduled onto precise VMs. Ineffective scheduling process leads to poor quality of service and leads to a large number of failures, which decreases trust in a cloud provider. Therefore, for preserving trust in the cloud provider while minimizing the rate of failures in the cloud paradigm, we proposed a fault-tolerant trust-aware task-scheduling algorithm (FTTATS). This approach initially considers the priorities of all tasks and VMs, and based on the collected priorities, the scheduler maps tasks to precise VMs. For the modelling of our proposed scheduler, we used Harris hawks optimization as a methodology which solves the task scheduling problem in a better way by not being trapped into a local optimum. FTTATS was implemented on Cloudsim and input for the algorithm was generated in two ways, i.e., by fabricating datasets with different distributions and indicated as D01, D02, D03, D04, and using real-time worklogs from HPC2N and NASA. Finally, the algorithm was compafred to state-of-the-art approaches, i.e., ACO, GA, and PSO. Results proved that FTTATS outperforms the existing approaches while minimizing makespan and rate of failures, as well as improving SLA-based trust parameters. The main limitation we observed in this research is the inability to predict the upcoming type of tasks, and therefore the scheduler is unable to identify and classify the tasks accurately. Therefore, in the future, a machine learning model can be employed in the scheduler to predict tasks upcoming onto the cloud console for an effective scheduling process in the cloud paradigm.

## Figures and Tables

**Table 1:** Summary of task-scheduling algorithms in the cloud paradigm.

Authors	Technique Used	Addressed Parameters
[7]	APSO	Makespan, throughput
[8]	LJ-PSO, M-PSO	makespan, total execution time, degree of imbalance
[9]	GAGELS	makespan, resource utilization
[10]	MPSO	Makespan, resource utilization
[11]	IT2FCM	Data movements, data placement, makespan
[12]	PSO-RDAL	Response time, task deadline, penalty cost
[13]	EPSOCHO	Makespan, processing cost, resource utilization
[14]	GSOS	Makespan, cost
[15]	AINN-BPSO	makespan, cost, degree of imbalance
[16]	QPSO	Scheduling efficiency
[17]	MVO-GA	Task transfer time
[18]	NSGAIII	runtime, cost, power consumption
[19]	Hybrid Lion-GA	Load balancing
[20]	GSAGA	Makespan
[21]	GBO	Makespan, accuracy of scheduling
[22]	HWOA-MBA	Makespan, cost
[23]	IWHOLF-TSC	Makespan, cost
[24]	HWACOA	Makespan, cost
[25]	LBACO	Datacenter processing time, response time, cost
[26]	QOGSHO	Makespan, resource utilization, consistency, SLA violations
[27]	ELHHO	Schedule length, execution cost, resource utilization
[28]	RATSA	Failure rate
[29]	SOATS	Cost, energy consumption
[30]	HunterPlus	Energy consumption, job completion rate
[31]	IQSSA	QOS parameters
[32]	RAO	Makespan
[33]	HFSGA	Makespan, cost
[34]	DRL	Makespan, throughput
[35]	IMOMVO	Execution time, throughput
[36]	HBSFD	Task processing time, turnaround time
[37]	Wale	Disk space
[38]	Docker Containers	Disk space

**Table 2:** Notations used in System Architecture.

Notation	Meaning
$ld^{vm^{n}}$	Workload on all considered VMs
$ld^{n}$	Workload on each VM
$ld^{h^{p}}$	Workload on all considered physical hosts
$po^{vm}$	Capacity of a VM
$to_{po}^{vm}$	Total capacity of all VMs
$t_{len}^{k}$	Length of all considered tasks
$t_{pri}^{k}$	Priorities of all considered tasks
$vm_{pri}^{j}$	Priorities of all considered VMs based on electricity cost
${d^{t}}^{k}$	Deadline constraint
$ex{e^{t}}^{k}$	Execution time of all considered tasks
$fi{n^{time}}^{k}$	Finish time for a task
$m^{k}$	Makespan of a task
RF	Rate of failures
$a(vm^{n})$	Availability of considered VMs
$SR(vm^{n})$	Success rate of considered VMs
$tt(vm^{n})$	Turnaround time of considered VMs
$tr^{CP}$	Trust in cloud provider

**Table 3:** Simulation Configuration Settings.

Name	Quantity
No. tasks	1000
Length of tasks	900,000
Memory of virtual host	2048 MB
Bandwidth of virtual resources	15 Mbps
Processing elements	1200 MIPS
Physical host memory	32 GB
Physical host hard disk capacity	2 TB
Bandwidth capacity of physical host	100 Mbps
Hypervisor type	Monolithic
Name of the hypervisor	Xen
OS of physical host	MAC
Operating system of virtual host	Linux
No. of datacenters	10

**Table 4:** Calculation of makespan using FTTATS.

No. of Tasks	ACO	GA	PSO	FTTATS
100 (D01)	708.12	715.43	692.34	612.43
500 (D01)	925.17	1338.26	1114.8	812.43
1000 (D01)	1412.45	1697.31	1824.6	921.37
**No. of Tasks**	**ACO**	**GA**	**PSO**	**FTTATS**
100 (D02)	952.18	921.39	830.17	727.5
500 (D02)	1323.71	1308.34	1419.18	931.26
1000 (D02)	1628.92	1698.13	1822.57	1308.21
**No. of Tasks**	**ACO**	**GA**	**PSO**	**FTTATS**
100 (D03)	872.43	742.56	837.28	621.53
500 (D03)	924.53	1298.21	1098.22	702.78
1000 (D03)	1413.7	1502.56	1267.87	953.12
**No. of Tasks**	**ACO**	**GA**	**PSO**	**FTTATS**
100 (D04)	624.98	711.28	624.78	509.32
500 (D04)	761.67	823.78	724.37	646.31
1000 (D04)	1412.76	1498.32	1331.27	1202.62
**No. of Tasks**	**ACO**	**GA**	**PSO**	**FTTATS**
100 (D05)	1472.1	1408.72	1873.16	821.37
500 (D05)	1784.6	2621.35	2187.23	1409.11
1000 (D05)	2653.98	3432.78	2821.11	1812.46
**No. of Tasks**	**ACO**	**GA**	**PSO**	**FTTATS**
100 (D06)	821.77	722.99	687.67	603.45
500 (D06)	931.45	1098.21	902.32	802.19
1000 (D06)	1421.76	1921.46	1902.32	1056.34

**Table 5:** Calculation of Rate of failures using FTTATS.

No. of Tasks	ACO	GA	PSO	FTTATS
100 (D01)	54.32	51.13	50.11	18.11
500 (D01)	63.52	60.29	59.37	22.13
1000 (D01)	45.14	47.14	48.22	17.11
**No. of Tasks**	**ACO**	**GA**	**PSO**	**FTTATS**
100 (D02)	51.67	48.43	46.78	21.15
500 (D02)	60.32	57.17	35.33	19.28
1000 (D02)	53.24	42.43	49.15	17.31
**No. of Tasks**	**ACO**	**GA**	**PSO**	**FTTATS**
100 (D03)	62.76	51.88	32.17	16.22
500 (D03)	45.17	48.37	27.32	24.37
1000 (D03)	56.18	38.21	20.17	14.29
**No. of Tasks**	**ACO**	**GA**	**PSO**	**FTTATS**
100 (D04)	45.31	63.88	71.56	18.13
500 (D04)	32.12	54.16	69.88	21.35
1000 (D04)	36.76	37.44	41.26	16.38
**No. of Tasks**	**ACO**	**GA**	**PSO**	**FTTATS**
100 (D05)	77.37	68.56	66.09	21.29
500 (D05)	69.87	71.22	71.43	18.98
1000 (D05)	71.23	75.32	61.33	24.17
**No. of Tasks**	**ACO**	**GA**	**PSO**	**FTTATS**
100 (D06)	56.34	68.32	56.87	19.72
500 (D06)	64.87	58.32	65.47	18.35
1000 (D06)	71.82	64.32	78.34	12.34

**Table 6:** Calculation of Availability of VMs using FTTATS.

No. of Tasks	ACO	GA	PSO	FTTATS
100 (D01)	65.21	67.88	71.24	86.87
500 (D01)	71.36	62.75	69.37	87.36
1000 (D01)	78.47	71.37	75.87	89.99
**No. of Tasks**	**ACO**	**GA**	**PSO**	**FTTATS**
100 (D02)	69.89	65.12	72.37	88.91
500 (D02)	72.77	59.88	67.17	82.99
1000 (D02)	65.44	61.17	74.88	90.37
**No. of Tasks**	**ACO**	**GA**	**PSO**	**FTTATS**
100 (D03)	77.32	58.47	68.09	85.44
500 (D03)	66.19	63.67	78.67	89.23
1000 (D03)	79.35	71.98	69.11	91.56
**No. of Tasks**	**ACO**	**GA**	**PSO**	**FTTATS**
100 (D04)	67.99	79.34	78.61	89.93
500 (D04)	72.43	82.11	62.19	91.65
1000 (D04)	76.97	69.14	79.98	94.35
**No. of Tasks**	**ACO**	**GA**	**PSO**	**FTTATS**
100 (D05)	52.47	59.45	54.12	82.56
500 (D05)	67.18	62.08	62.18	89.17
1000 (D05)	74.86	67.44	69.23	90.12
**No. of Tasks**	**ACO**	**GA**	**PSO**	**FTTATS**
100 (D06)	42.37	54.32	62.21	87.42
500 (D06)	54.65	61.12	71.88	91.26
1000 (D06)	63.18	69.98	67.10	94.31

**Table 7:** Calculation of Success rate of VMs using FTTATS.

No. of Tasks	ACO	GA	PSO	FTTATS
100 (D01)	72.17	69.21	71.24	87.19
500 (D01)	62.34	75.32	62.17	91.35
1000 (D01)	49.36	79.21	59.61	96.36
**No. of Tasks**	**ACO**	**GA**	**PSO**	**FTTATS**
100 (D02)	54.61	59.57	74.88	87.38
500 (D02)	61.37	64.32	80.19	90.14
1000 (D02)	69.57	71.37	82.17	94.66
**No. of Tasks**	**ACO**	**GA**	**PSO**	**FTTATS**
100 (D03)	64.89	55.31	73.16	89.09
500 (D03)	72.61	61.16	80.11	93.46
1000 (D03)	65.15	69.16	84.57	96.17
**No. of Tasks**	**ACO**	**GA**	**PSO**	**FTTATS**
100 (D04)	56.39	58.81	75.20	84.16
500 (D04)	60.47	68.16	62.11	91.98
1000 (D04)	70.12	72.39	59.26	97.15
**No. of Tasks**	**ACO**	**GA**	**PSO**	**FTTATS**
100 (D05)	48.15	62.26	57.87	90.29
500 (D05)	55.06	68.67	67.31	94.37
1000 (D05)	61.02	73.22	74.36	98.51
**No. of Tasks**	**ACO**	**GA**	**PSO**	**FTTATS**
100 (D06)	46.44	52.21	66.43	88.21
500 (D06)	59.11	63.76	76.06	95.18
1000 (D06)	66.29	74.07	84.01	98.29

**Table 8:** Calculation of turnaround efficiency using FTTATS.

No. of Tasks	ACO	GA	PSO	FTTATS
100 (D01)	64.28	53.12	53.19	88.36
500 (D01)	69.96	61.27	65.28	90.94
1000 (D01)	51.02	67.16	60.37	97.54
**No. of Tasks**	**ACO**	**GA**	**PSO**	**FTTATS**
100 (D02)	57.37	58.66	62.18	85.87
500 (D02)	62.49	60.97	71.19	91.27
1000 (D02)	70.17	73.59	75.37	96.29
**No. of Tasks**	**ACO**	**GA**	**PSO**	**FTTATS**
100 (D03)	61.33	58.13	61.18	87.11
500 (D03)	64.19	65.10	77.26	94.48
1000 (D03)	69.26	74.16	69.19	97.28
**No. of Tasks**	**ACO**	**GA**	**PSO**	**FTTATS**
100 (D04)	56.57	53.27	71.51	86.16
500 (D04)	65.36	62.03	82.27	95.12
1000 (D04)	72.43	69.18	83.68	97.38
**No. of Tasks**	**ACO**	**GA**	**PSO**	**FTTATS**
100 (D05)	45.15	60.19	59.36	91.17
500 (D05)	52.46	63.46	63.39	95.29
1000 (D05)	59.15	71.58	76.12	98.09
**No. of Tasks**	**ACO**	**GA**	**PSO**	**FTTATS**
100 (D06)	56.17	55.39	72.19	90.06
500 (D06)	64.38	60.35	79.25	94.22
1000 (D06)	68.87	70.78	82.27	98.78

**Table 9:** Improvement of makespan for FTTATS over existing algorithms.

Dataset	ACO	GA	PSO
D01	22.96	37.45	35.39
D02	24.01	24.45	27.13
D03	29.06	35.72	28.9
D04	15.75	22.25	12.01
D05	31.6	45.82	41.25
D06	22.45	34.21	29.5

**Table 10:** Minimization of Rate of Failures for FTTATS over existing algorithms.

Dataset	ACO	GA	PSO
D01	64.81	63.84	63.64
D02	65.06	61.01	56.02
D03	66.56	60.36	31.11
D04	51.07	64.06	69.42
D05	70.5	70.04	67.59
D06	73.88	73.6	74.88

**Table 11:** Improvement of Availability of VMs for FTTATS over existing algorithms.

Dataset	ACO	GA	PSO
D01	22.86	30.80	22.04
D02	26.03	40.88	22.31
D03	19.46	37.15	23.33
D04	26.92	19.65	24.97
D05	34.62	38.56	41.13
D06	70.39	47.23	35.68

**Table 12:** Improvement of Success rate of VMs for FTTATS over existing algorithms.

Dataset	ACO	GA	PSO
D01	49.5	22.86	42.41
D02	46.67	39.46	14.71
D03	37.52	50.15	17.17
D04	46.16	37.08	39.02
D05	72.43	38.7	41.91
D06	63.91	48.23	75.5

**Table 13:** Improvement of turnaround efficiency of VMs for FTTATS over existing algorithms.

Dataset	ACO	GA	PSO
D01	49.44	52.4	54.80
D02	43.89	41.52	30.98
D03	43.17	41.28	34.3
D04	43.37	51.04	17.34
D05	81.53	45.76	43.08
D06	49.42	51.76	21.11

## Data Availability

Authors are not interested in disclosing the data.
